# Preclinical *in vitro* and *in vivo* evaluation of omadacycline against multidrug-resistant *Acinetobacter baumannii*

**DOI:** 10.1128/aac.00932-25

**Published:** 2025-10-30

**Authors:** Pattaraporn Vanachayangkul, Nattaya Ruamsap, Diana Caridha, Rawiwan Imerbsin, Erin Ball, Supaksorn Chattagul, Anchalee Tungtang, Siriphan Gonwong, Chanikarn Kodchakorn, Paphavee Lertsethtakarn, Sasikorn Silapong, Chaiyaporn Chaisatit, Nuanpan Khemnu, Daniel Boudreaux, Nonlawat Boonyalai, Samandra Demons, Jessica V. Pierce, Monica Martin, Diane Anastasiou, Alisa W. Serio, Brian Vesely, Charlotte Anne Lanteri

**Affiliations:** 1 Department of Bacterial and Parasitic Diseases, Walter Reed Army Institute of Research-Armed Forces Research Institute of Medical Science 19965 , Bangkok, Thailand; 2 Experimental Therapeutics Branch, Walter Reed Army Institute of Research 8394, Silver Spring, Maryland, USA; 3 Department of Veterinary Medicine, Walter Reed Army Institute of Research-Armed Forces Research Institute of Medical Science 19965 , Bangkok, Thailand; 4 Paratek Pharmaceuticals Inc., King of Prussia, Pennsylvania, USA; University of Fribourg, Fribourg, Switzerland

**Keywords:** *A. baumannii*, omadacycline, murine models, PK/PD, wound infection, multi-drug resistant, antibiotic

## Abstract

*Acinetobacter baumannii* is an increasing cause of chronic wound infections, especially in military operational settings, and is resistant to many currently available antimicrobial therapies, making treatment options limited. We evaluated the *in vitro* activity of omadacycline against *A. baumannii* clinical isolates as well as the *in vivo* efficacy of omadacycline in mice against the multi-drug resistant (MDR) *A. baumannii* strain 5075 using both a neutropenic thigh infection model and a dorsal wound model of chronic infection relative to the standard of care drugs tigecycline and doxycycline, respectively. Omadacycline demonstrated efficacy against these two established preclinical models of MDR *A. baumannii* infection in mice. In the dorsal wound infection model, the most efficacious dose tested (10 mg/kg twice daily for 6 days) resulted in 100% survival, complete clearance of systemic infection, accelerated wound closure, absence of toxicity, significantly reduced inflammatory cytokine responses, and reduced biofilm formation. These results underscore the potential of omadacycline as a promising therapeutic option for combating MDR *A. baumannii* infections in chronic wound settings and that additional research is warranted.

## INTRODUCTION

Antimicrobial resistance (AMR) continues to spread globally, limiting treatment options and accounting for almost 5 million deaths annually in 2019 (1). A recent report from 2019 estimated that AMR is the third greatest contributor to the global burden of disease; only ischemic heart disease and stroke had higher mortality rates (2). *Acinetobacter baumannii* is an opportunistic gram-negative bacterial pathogen that can cause infection in the blood, lungs, and urinary tract, as well as wounds throughout the body (3). Furthermore, *A. baumannii* frequently forms robust biofilms on skin and soft tissues including on wounds and occlusive dressings (4). Biofilm formation significantly contributes to the increased virulence and antibiotic resistance because it enhances (i) the reduced diffusion of antibiotics into biofilm via the produced extracellular matrix as a protective barrier, (ii) the horizontal transfer of antibiotic resistance genes between bacterial cells within biofilm compartment, and (iii) mutation at high rate due to cell stress (5). The Centers for Disease Control and Prevention reported an increasing incidence of *A. baumannii* infections associated with US Army service members injured during Operation Iraqi Freedom and Operation Enduring Freedom from 2002 to 2004 (6). In addition, in 2017, carbapenem-resistant *A. baumannii* caused an estimated 8,500 infections in hospitalized patients and 700 estimated deaths in the United States (US) with an estimated healthcare cost of $280M (6). Human conflict is a significant driver of AMR with global consequences for healthcare systems. In the case of Ukraine, individuals affected by conflict who are forced to travel and become internationally displaced—living in shelters and crowded settings—enhance the risk of person-to-person transmission of infections, including infections that may be caused by drug-resistant pathogens (7). In 2023, McGann et al. reported growth of six extensively drug-resistant bacterial strains from blood and surveillance cultures (*A. baumannii*, *Enterococcus faecium*, *Klebsiella pneumoniae*, and three distinct *Pseudomonas aeruginosa* strains) from a mid-50s age injured service member deployed to Ukraine. All of the gram-negative pathogens isolated were non-susceptible to almost every antibiotic tested, except for *A. baumannii*, which was susceptible to cefiderocol, colistin, and tetracycline class drugs, including eravacycline and omadacycline (8).

Omadacycline, an aminomethylcycline within the tetracycline class and semi-synthetic derivative of minocycline, was approved by the US Food and Drug Administration (FDA) in 2018 for the treatment of adults with acute bacterial skin and skin structure infections (ABSSSIs) and community-acquired bacterial pneumonia with both intravenous (IV) and oral formulations (9–12). Omadacycline has broad-spectrum activity including against certain gram-positive, gram-negative, anaerobic, and atypical pathogens (13). Mechanistically, omadacycline exerts its antimicrobial effect by inhibiting bacterial protein synthesis via binding to the 30S subunit of the bacterial ribosome. Through structural modifications of the tetracycline core, omadacycline is designed to overcome two major tetracycline-class resistance mechanisms: efflux pumps and ribosomal protection proteins (14).

In this study, we evaluated the *in vitro* activity of omadacycline and comparator drugs against clinical isolates of *A. baumannii*. In addition, we performed *in vivo* evaluations of omadacycline efficacy in mice against multi-drug resistant (MDR) *A. baumannii* strain 5075 in both a neutropenic thigh and dorsal wound infection model. This research aimed to explore omadacycline’s potential against challenging infections, specifically for combat wound infections caused by *A. baumannii*.

## MATERIALS AND METHODS

### *In vitro* antimicrobial susceptibility testing

#### Bacterial strains and *in vitro* growth conditions

The activity of omadacycline and standard-of-care comparator antibiotics was evaluated against 100 genotypically diverse and broadly drug-resistant *A. baumannii* from the Walter Reed Army Institute of Research (WRAIR) Multidrug-Resistant Organism Repository and Surveillance Network (MRSN) (14). This diversity panel was constructed from over 3,500 clinical isolates collected from various infection types (e.g., wound, respiratory, blood, and urine) between 2003 and 2017 from Military Health System (MHS) patients and other surveillance sources in the United States, Europe, Central America, South America, and Asia (15). Genetic sequencing of the isolates was performed and confirmed the presence of diverse AMR genes including aminoglycoside modifying enzymes, beta-lactamases, carbapenemases, and tetracycline resistance genes.

Quality control (QC) testing was performed using *Escherichia coli* American Type Culture Collection (ATCC) 25922 and *S. aureus* ATCC 29213 (ATCC, VA, USA) for omadacycline and the standard-of-care comparator antibiotics as recommended by the Clinical and Laboratory Standards Institute (CLSI) guidelines. Levofloxacin MICs were previously determined, and this drug was included as an internal assay control (16). All MIC values obtained for the QC strains were within the acceptable CLSI approved QC ranges.

Bacterial strains were initially plated on blood agar with sheep blood (Thermo Fisher Scientific, MA, USA) from freezer stocks and incubated overnight at 37°C. Colony-forming units (CFU) from each strain were suspended into separate vials of 5 mL demineralized water (Thermo Fisher Scientific, MA, USA) with a sterile cotton swab and vortexed. A nephelometer (Thermo Fisher Scientific, MA, USA) calibrated with a 0.5 McFarland Standard (Remel, KS, USA) was used to determine the appropriate concentration of cells. When the desired turbidity was reached, 10 µL of each bacterial cell suspension was placed into 11 mL of cation-adjusted Mueller-Hinton broth (CAMHB) (Thermo Fisher Scientific, MA, USA) and vortexed to yield a starting inoculum of 1 × 10^5^ CFU/mL and was subsequently transferred aseptically to a sterile deep 96-well plate using the TECAN Freedom EVO200 Liquid Handling Robot (Tecan, Switzerland) along with CAMHB-only controls.

#### Determination of MICs

MIC assays were performed in duplicate. Ninety-six-well microtiter plates with twofold serial dilutions of antibiotics with a final concentration range of 128–0.25 µg/mL with an assay volume of 100 µL (TECAN Freedom EVO200 Liquid Handling Robot) were utilized. Following inoculation, plates were incubated at 37°C for 20 hours without agitation, and MIC values were read and recorded using BIOMIC V3 digital imaging system (Giles Scientific, CA, USA) to compare turbidity and image data from each test well to growth and no-growth control wells (17). The lowest antibiotic concentration that inhibited visible growth indicated the MIC. The results and corresponding images were then automatically recorded and exported into a Microsoft Excel file to determine the MIC_50_ and MIC_90_ of each bacterial isolate.

Omadacycline (Paratek Pharmaceuticals, Inc., PA, USA) and the standard-of-care comparator antibiotics including doxycycline (Sigma-Aldrich, MO, USA), tigecycline (Sigma-Aldrich, MO, USA), and internal positive control levofloxacin (United States Pharmacopeia, MD, USA) were solubilized in either water or dimethyl sulfoxide.

### Animal models

#### Ethics statements for animal work

Animal research was conducted at an AAALAC accredited facility in compliance with the Animal Welfare Act and other federal statutes and regulations relating to animals and experiments involving animals and adheres to principles stated in the *Guide for the Care and Use of Laboratory Animals*, NRC Publication, 2011 edition (18). Two animal use protocols (AUPs) were performed independently. Each protocol was approved by an Institutional Animal Care and Use Committee at WRAIR and Armed Forces Research Institute of Medical Sciences (AFRIMS), respectively.

For WRAIR AUP, male and female ICR mice (*Mus musculus*, age 4–6 weeks) were purchased from Charles River Laboratories (Wilmington, MA, USA), and for AFRIMS AUP, female ICR mice (10–14 weeks) were bred, raised, and maintained at the Department of Veterinary Medicine, AFRIMS. The mice were acclimatized for 7 days and randomly assigned a study number. Mice were housed in a designated room with food and water supplied *ad libitum* and a 12:12 hours light:dark cycle.

#### Drug preparation for *in vivo* studies

Omadacycline, 100 mg/vial for injection, was obtained from Paratek Pharmaceuticals, Inc. (USA). Cyclophosphamide monohydrate was obtained from Baxter Healthcare Corporation (Deerfield, IL, USA) for WRAIR AUP and from Baxter Oncology GmbH (Halle Westphalia, Germany) for AFRIMS AUP. Doxycycline hyclate and tigecycline were purchased from Sigma-Aldrich (MO, USA). Cyclophosphamide, tigecycline, and omadacycline were prepared in sterile 0.9% sodium chloride, and doxycycline was dissolved in sterile phosphate buffered saline (PBS) for *in vivo* dosing aseptically.

#### Neutropenic mouse thigh infection model of *A. baumannii* 5075 (WRAIR AUP)

The MDR *A. baumannii* strain AB5075 was used for animal infections (19). Inoculums for mouse infection were prepared from an overnight culture in Luria-Bertani (LB) broth (Thermo Fisher Scientific, MA, USA) with 100 µg/mL ampicillin (to maintain the plasmid) (Sigma-Aldrich, MO, USA) at 37°C with agitation (250 rpm) and then were sub-cultured (37°C with agitation [250 rpm]) and grown to exponential phase (~1 hour). Exponential phase cells were then pelleted at 5,000 rpm for 5 min, washed three times with 1 mL of sterile saline solution (Baxter International, IL, USA), and diluted to the final target inoculum dose of 1 × 10^6^ CFU in 25 µL. To verify the inoculation dose, the inoculum was serially diluted 10-fold, and 100 µL of each dilution was plated onto duplicate LB agar plates before overnight incubation at 37°C and CFU enumeration.

A total of 10 ICR mice, five males and five females, with an average weight of 31.02 grams were assigned to each study group. Briefly, two doses of cyclophosphamide, 150 mg/kg and 100 mg/kg, were administered intraperitoneally on days (−4) and (−1) prior to bacterial inoculation, respectively. Anesthetized mice were infected using insulin 28G 1/2” syringes (catalog #26026, lot# 230425, EXELINT International, Co. CA, USA) with 10^6^ CFU contained in a 25 µL inoculum of *A. baumannii* strain 5075 bacteria *via* intramuscular injection into the thigh. Treatment of mice with subcutaneous dosing of omadacycline, comparator control standard-of-care antibiotic tigecycline, or infection control (sterile 0.9% sodium chloride) occurred 2 hours post-animal infections, the time in which the bacterial load in the infected thigh is increasing exponentially. Omadacycline, tigecycline, and the infection control were given subcutaneously (SC) at 10 and 25 mg/kg, twice a day (BID) for 1 day with a maximum volume of 100 µL. Mice were euthanized 24 hours post-infection and infected thighs were harvested, weighed, and homogenized using a TissueRuptor II Homogenizer (Catalog # 9002755, Qiagen Sciences Inc, MD, USA) in 2 mL of ice-cold, sterile PBS prior to plating on Omni-plate LB agar to determine CFU/g for each sample to determine bacterial tissue burden.

#### Mouse dorsal wound model of *A. baumannii* 5075 infection (AFRIMS AUP)

Thirty ICR female mice with an average weight of 40.6 grams (measured on day 4), and 40 ICR female mice with an average weight of 43.6 grams (measured on day 4) were assigned to each study group of cohorts 1 and 2, respectively. In cohort 1, a total of 30 ICR mice (*n* = 5/group) were divided into six groups; group 1 (infection control), group 2 (doxycycline 25 mg/kg), group 3 (omadacycline 5 mg/kg), group 4 (omadacycline 10 mg/kg), group 5 (omadacycline 20 mg/kg), and group 6 (omadacycline 25 mg/kg). The doses that most effectively improved wound healing and cleared bacteria in wounds and systemic organs were selected for evaluation in cohort 2. In cohort 2, a total of 40 ICR mice (*n* = 10/group) were randomly assigned to four groups; group 1 (infection control), group 2 (doxycycline 25  mg/kg), group 3 (omadacycline 10 mg/kg), and group 4 (omadacycline 15 mg/kg).

The MDR *A. baumannii* AB5075 clinical isolate was utilized as the challenge strain (19). Inoculum for wound infection was similar to that as previously described above except that the bacterial suspension was adjusted to a final concentration of 5.0 × 10^4^ CFU in 25 µL of PBS for wound inoculation.

On day 0 before wounding, mice were anesthetized by IM injection with a combination of 40 mg of ketamine, 2 mg of xylazine, and 0.06  mg of atropine (0.1–0.2 mL/100 g body weight). All hair on the back from the cervical to m-d lumbar region was removed and the skin was disinfected. A full-thickness dorsal wound on each side of the midline was created using a 6.0 mm disposable skin biopsy punch (VisiPunch; Huot Instruments, WI, USA). Each wound was inoculated with 5  ×  10^4^ CFU of *A. baumannii* 5075 in 25 µL PBS and then allowed to absorb for 3 min. Transparent wound dressings (3M Tegaderm, MN, USA) were applied over each wound site. All treatments were administered *via* intraperitoneal (IP) injection, BID from days 0–5, with the first treatment dose given at 4 hours-post-infection. Mice were SC injected with 0.1  mg/kg of buprenex, BID, to reduce pain.

Three mice from each group were euthanized on day 7, while the remaining two mice were euthanized on day 21 in order to collect tissue samples from the wound bed, spleen, liver, and lung to determine bacterial burden. Mice from all groups of a second cohort were euthanized as follows: day 3 (*n* = 2), day 7 (*n* = 3), and day 21 (*n* = 5) to collect tissue samples from the wound bed and organs as well as whole blood and Tegaderm dressings to determine bacterial burden, cytokine responses, and biofilm disruption, respectively. A clinical scoring system was utilized to assess clinical signs of disease as outlined in the approved protocol to assess for signs of disease.

#### Tissue burden

Following euthanasia, wound beds, spleens, livers, and lungs were collected aseptically to quantify viable bacteria by CFU. After collection, wound beds and organs were rinsed several times with PBS and weighed. Tissue homogenates were obtained by placing the collected tissues inside a cell strainer (Becton, Dickinson, MD, USA), which was then placed on a sterile petri dish, and then the tissues were ground using a syringe plunger to achieve homogenization. The homogenates were 10-fold serially diluted and 100 µL was plated on eosin methylene blue agar (Becton, Dickinson, MD, USA), followed by an overnight incubation at 37°C to determine the CFU/g tissue.

#### Cytokine measurement

Whole blood via cardiac puncture and tail from the euthanized and viable mice, respectively, was collected in ethylenediaminetetraacetic acid vacutainer tubes and plasma was separated by centrifugation at 1,000 × *g* for 10 min at 25°C. Plasma was collected using a single-channel pipette and then transferred to another sterile microcentrifuge tube followed by re-centrifugation to remove any particulates. Separated plasma was collected and stored at −70±5°C.

Pro-inflammatory cytokines/chemokines (IL-6, TNF-α, and MCP-1) were quantitatively measured using Merck Millipore’s Milliplex Mouse Cytokine/Chemokine Magnetic Bead kit (Merck Millipore, Germany) per the manufacturer’s instructions. Data acquisition was performed on the Luminex MAGPIX system (Luminex, TX, USA) and cytokine levels were analyzed using xPONENT software (Luminex, TX, USA).

#### Histopathologic evaluation of wounds

Tissue samples were collected from the dorsal wound bed, fixed in 10% neutral buffered formalin solution (Sigma-Aldrich, MO, USA) for 24–72 hours, and processed using an automated tissue processor (Leica Biosystems Imaging PT1020, Nussloch, Germany). Tissues were embedded in paraffin blocks, sectioned at 5 µm using a semi-automated rotary microtome (RM2245, Leica, IL, USA), mounted onto poly L-lysine coated microscope slides, baked at 60°C for 14–18 hours, and stained with hematoxylin-eosin (H&E). Slides were evaluated by a blinded board-certified veterinary anatomic pathologist. H&E-stained slides were scanned at 20× magnification using an Aperio slide scanner (Leica Biosystems Imaging, CA, USA) with a magnification doubler and a resolution of 0.505 mm/pixel. Minimal contrast, color, and white balance adjustments/corrections were made (using Adobe Photoshop) to reveal tissue detail and facilitate identification of microscopic lesions.

A total of 48 wound biopsies from mice were examined microscopically and via histopathologic severity scores based on the known progression of tissue repair and results from previous experiments (20). The histopathologic criteria evaluated for each wound biopsy included ulceration, presence of bacterial colonies, inflammation, muscle necrosis, and fibrosis. While efforts were made to section the central area of the wound for histopathology in all cases, the process of fixation, embedding, and microtome sectioning introduced the possibility of sample variation. As lesions often extended beyond the deep margin of the biopsy, wound depth was not evaluated.

#### SEM of Tegaderm wound dressings

The formation of mature biofilm was observed by scanning electron microscopy (SEM) (21, 22). Tegaderm dressings were carefully collected from the wounds without folding at 1, 3, and 7 days post-infection (dpi). The collected Tegaderm was adhered to aluminum foil by facing bacteria upward, and the base of aluminum foil was affixed onto a 12 mm round glass coverslip followed by placing the samples in the well of 24-well tissue culture plate. After that, 1 mL of fixative reagent (1% glutaraldehyde and 2.5% paraformaldehyde in sterile PBS) was added to each sample well. To prevent evaporation, 1 mL of sterile PBS was added to peripheral wells. The fixed Tegaderm was stored in a 2°C–8°C refrigerator.

Fixed Tegaderm wound dressings were viewed by SEM to capture bacterial biofilm images (performed at the Department of Pathology, Faculty of Tropical Medicine, Mahidol University). Samples were scanned at magnifications ranging from 1,000 to 10,000× within the Tegaderm wound dressings. Representative photomicrographs showing biofilm formation were captured at 5,000× magnification.

#### 16S rRNA sequencing for wound microbiome

DNA was extracted from 25 to 30 mg of wound bed using DNeasy Blood and Tissue Kit (Qiagen, Hilden, Germany) with modifications. The tissue was lysed with 230 µL of QiaAmp ATL and 20 µL of proteinase K in seven to eight beads of 2.0 mm zirconium silicate beads. The mixture was beaten for 3 min and incubated at 56°C until the tissue was completely lysed and DNA was isolated per the manufacturer’s instruction.

DNA purity and yield were determined by NanoDrop 2000/200C Spectrophotometer (Thermo Fisher Scientific, DE, USA) and Qubit 4 Fluorometer with dsDNA HS Assay kit, respectively. The DNA was sequenced commercially by Novogene (China). For sequencing, 16S rRNA was amplified using 16SV3-V4 primer sets with a barcode in a 15 µL reaction using Phusion High-Fidelity PCR Master Mix (New England Biolabs, MA, USA) using 10 ng of DNA per sample. The PCR products were visualized with SYBR Green on 2% agarose electrophoresis gel. The PCR products were mixed in equidensity ratios and purified with Universal DNA Purification Kit (TianGen, China). Sequencing libraries were generated using NEB Next Ultra II FS DNA PCR-free Library Prep Kit (New England Biolabs, MA, USA). The libraries were quantified using Qubit and real-time PCR. Quantified libraries were then pooled and sequenced on the Illumina platform.

The resulting sequence data set was denoised using DADA2 (Li et al., 2020) and initial amplicon sequence variants (ASVs) were generated. The obtained ASVs were used to annotate species using QIIME2 software and queried in the Silva Database. The absolute abundance of ASVs was normalized to determine relative abundance. Alpha diversity was calculated in QIIME2 using the following factors: Chao1 and Shannon. Unweighted and weighted distances were used to calculate dissimilarity coefficients between pairwise samples.

#### Statistical analysis

Statistical analysis was conducted using GraphPad Prism version 9.4.1 software package (GraphPad Software, Inc., CA, USA). One-way ANOVA with Dunnett’s multi-comparison test and an unpaired *t*-test with Welch’s correction were used to compare the differences of bacterial CFUs, wound area, body weight, cytokine levels, and histopathological scores between groups. A *P* < 0.05 was considered statistically significant.

## RESULTS

### *In vitro* antimicrobial susceptibility testing

#### *A. baumannii* clinical isolate diversity panel

**Fig 1:**
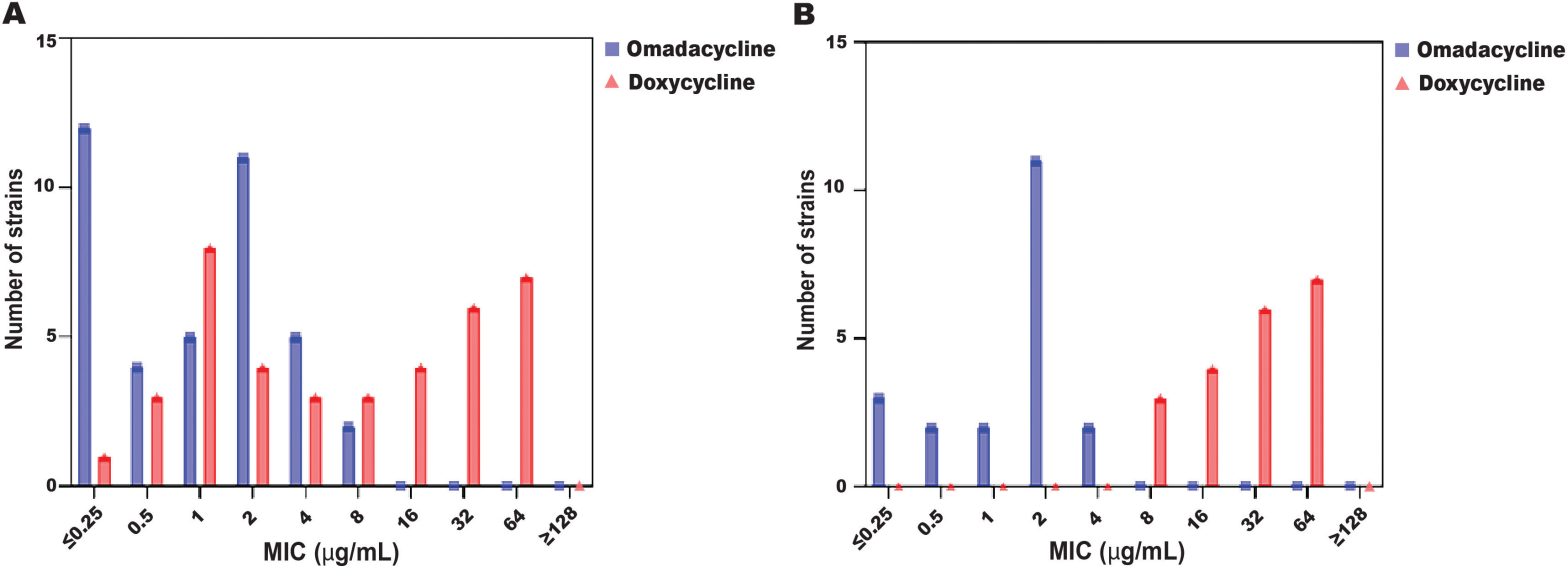
A histogram represents the MIC distribution of omadacycline and doxycycline against tetracycline-resistant *A. baumannii* isolates (*N* = 39) (**A**) and against doxycycline-resistant *A. baumannii* isolates (*N* = 20) (**B**). Each bar represents the number of bacteria isolates exhibiting a specific MIC value for each antibiotic tested.

Omadacycline exhibited potent *in vitro* activity against geographically, genetically, and phenotypically diverse *A. baumannii* clinical isolates. The *in vitro* activity of omadacycline (MIC_50_ 0.25 µg/mL, MIC_90_ 2 µg/mL) was on par with that of tigecycline (MIC_50_ 0.25 µg/mL, MIC_90_ 1 µg/mL) and slightly better than doxycycline (MIC_50_ 0.25 µg/mL, MIC_90_ 32 µg/mL) (Table 1). The highest MIC value observed for omadacycline was 8 µg/mL against two isolates, which were susceptible to all other comparator drugs (Table S1). The MIC distribution analysis revealed that omadacycline exhibited lower MIC values compared to doxycycline against both tetracycline-resistant and doxycycline-resistant *A. baumannii* isolates (Fig. 1A and B). Among tetracycline-resistant isolates (Fig. 1A), the majority showed MIC values of 1–4 µg/mL for omadacycline, while doxycycline MICs were generally higher, ranging from 4 to >16 µg/mL. Similarly, in doxycycline-resistant isolates (Fig. 1B), omadacycline maintained lower MICs, suggesting retained activity, whereas doxycycline MICs clustered at higher levels, indicating reduced susceptibility.

**TABLE 1:** *In vitro* activity of omadacycline and comparator antibiotics against *A. baumannii* clinical isolates (*N* = 100)

Antibiotic agent	MIC range (µg/mL)	MIC (µg/mL)	
		MIC_50_	MIC_90_
Omadacycline	≤0.25–8	0.25	2
Doxycycline	≤0.25–64	0.25	32
Tigecycline	≤0.25–8	0.25	1
Levofloxacin	≤0.25–128	4	16

### Neutropenic mouse thigh model of *A. baumannii* 5075 infection

**Fig 2:**
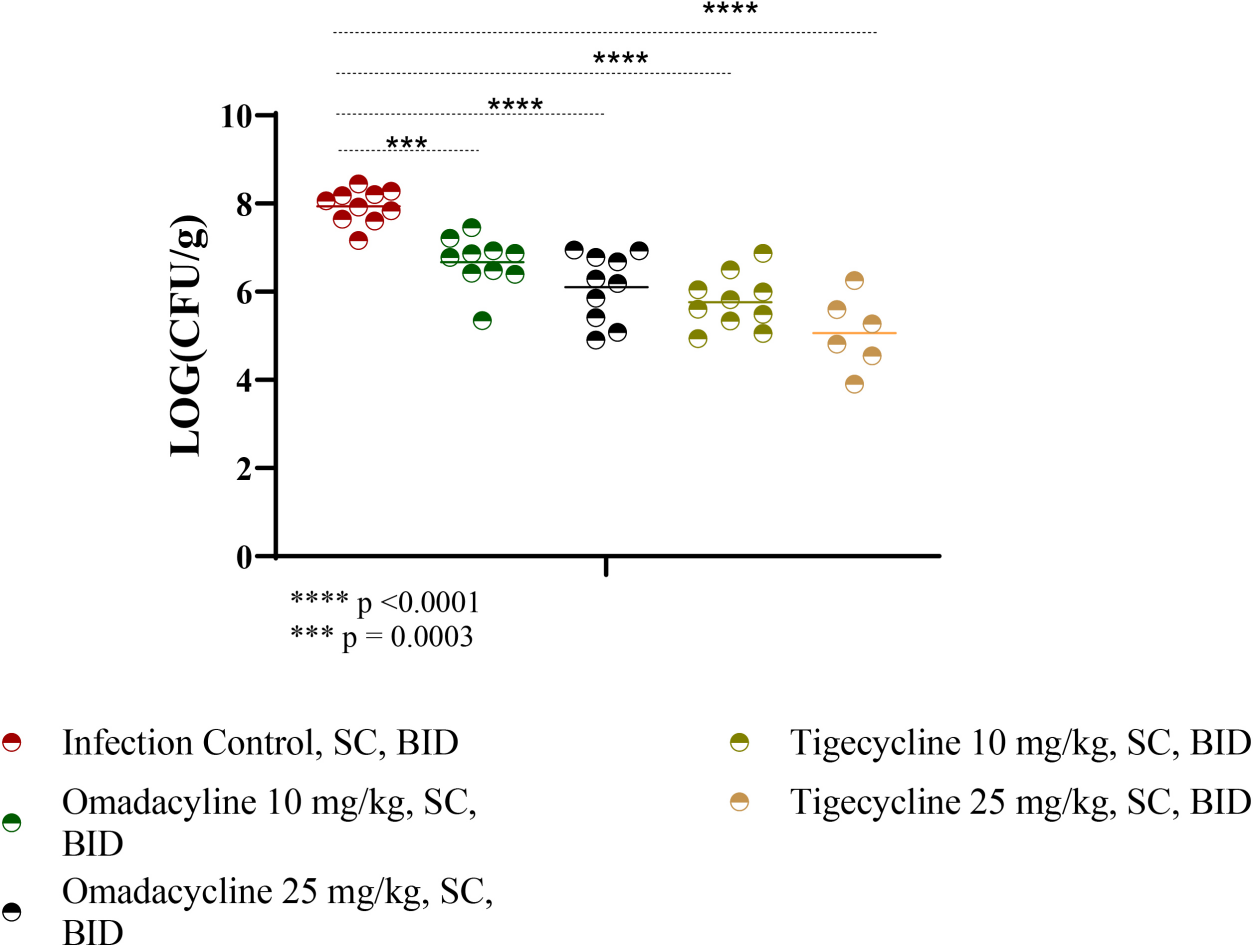
Omadacycline and tigecycline efficacy in neutropenic ICR mice infected with *A. baumannii.* ICR mice were infected via intramuscular injection and administered omadacycline or tigecycline at 10 and 25 mg/kg or infection control SC, BID. Infected thighs were collected 24 hours post-infection, *** *P* = 0.0003 and **** *P* < 0.0001.

At twice daily SC administered doses of 10 and 25 mg/kg, both omadacycline and tigecycline significantly reduced the bacterial loads of *A. baumannii* strain 5075 compared to the infection control in the thighs of neutropenic ICR mice (Fig. 2). Compared to the infection control, reductions of 1.26 (*P* = 0.0003), 1.82 (*P* < 0.0001), 2.16 (*P* < 0.0001), and 2.87 (*P* < 0.0001) log 10 colony forming units per gram (log10 CFU/g) were observed, respectively, for omadacycline 10 mg/kg, omadacycline 25 mg/kg, tigecycline 10 mg/kg, and tigecycline 25 mg/kg treatment regimens. Notably, there was no significant difference between the bacterial load of omadacycline at 25 mg/kg and tigecycline at 10 mg/kg.

### *In vivo* efficacy of omadacycline in a murine dorsal wound model

#### Survival

The 21-day survival rates for *A. baumannii*-infected mice (cohort 1) treated for 6 days with omadacycline 5, 10, 20, or 25 mg/kg (IP, two times daily) were 40%, 100%, 60%, 80%, respectively (Fig. 2A). All (100%) of the mice treated with doxycycline 25 mg/kg survived, while only 40% of placebo-treated mice (infection control) survived. The survival rates demonstrated with doxycycline (25 mg/kg) and omadacycline (10 mg/kg) treatments were statistically different than that of the infection control (*P* < 0.05), but there was no statistical difference observed for the other omadacycline doses evaluated compared to the infection control group.

The omadacycline 10 mg/kg group had higher survival rates compared to the omadacycline 20 and 25 mg/kg groups, and mice treated with these higher doses of omadacycline also had signs of discomfort after IP injection as demonstrated by presenting hunchback behavior (Fig. 2A). Based on the observed efficacy, omadacycline doses of 10 mg/kg and 15 mg/kg were selected for evaluation in cohort 2.

In cohort 2, *A. baumannii*-infected mice that received treatment with doxycycline 25 mg/kg and omadacycline 10 mg/kg and 15 mg/kg had higher survival rates (100%, 100%, and 90%, respectively) compared to the infection control group (70%) (Fig. 2B), but were not significantly different. The 100% survival demonstrated in the omadacycline 10 mg/kg treatment group was identical to the previous observation in cohort 1. The percent survival of mice in the infection control groups differed between cohort 1 and 2: survival rate of cohort 1 (40% survival; 3/5 mice died) was lower than that of cohort 2 due to the larger group size (70% survival; 3/10 mice died).

**Fig 3:**
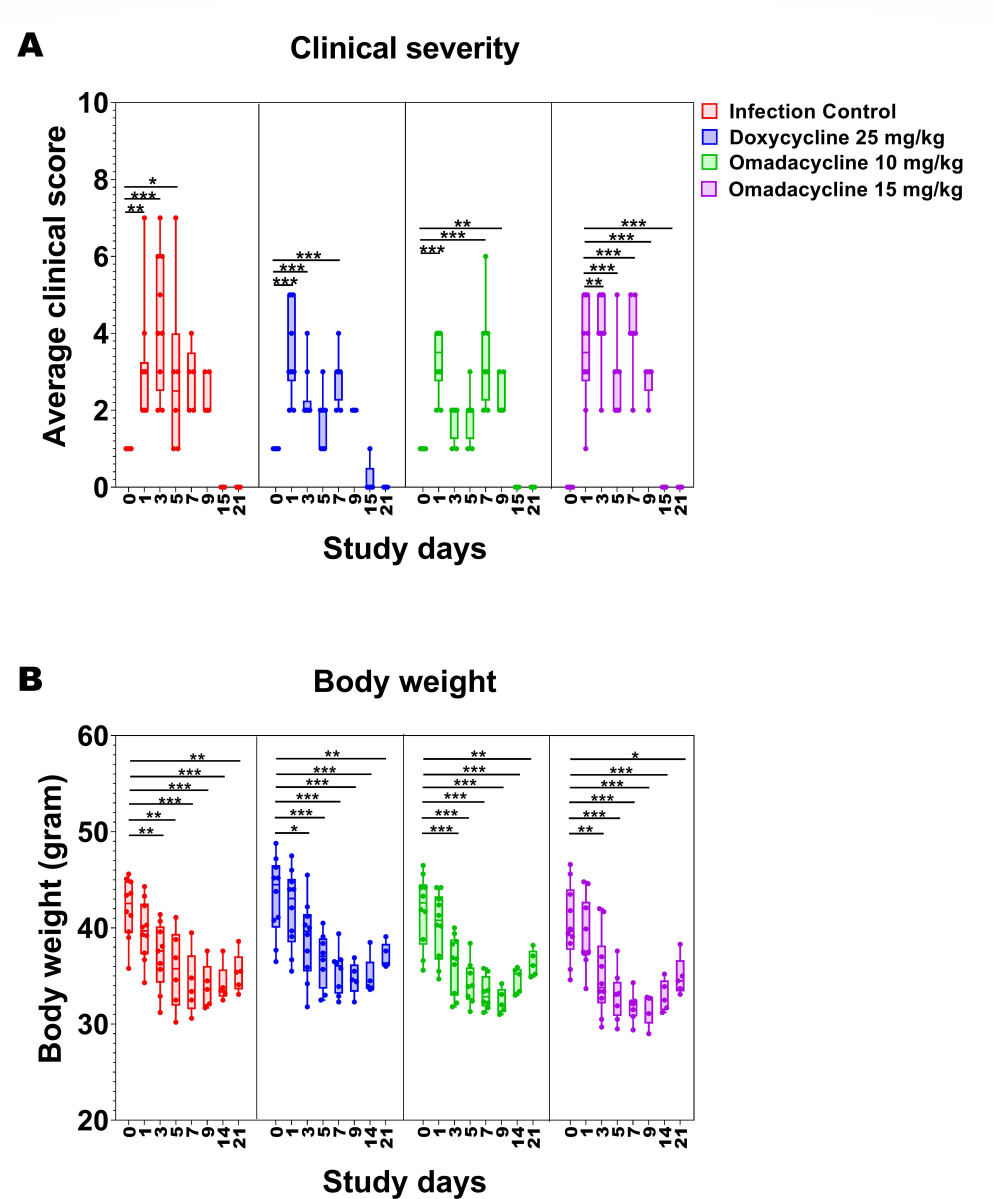
Cohort 2: Clinical scores (**A**) and body weight (**B**) in *A. baumannii*-infected mice treated with either doxycycline or omadacycline. Each dot represents data from one mouse. The box-and-whisker plots show the median and interquartile range with 95% CI. *P*-values show significant differences compared to day 0 within the same group (one-way ANOVA followed by Dunnett’s multiple comparisons test; *, *P* < 0.05; **, *P* < 0.01; ***, *P* < 0.001).

Clinical scores indicating disease were observed for all animals during the study in both cohorts 1 and 2. As shown in Fig. 3A, clinical scores peaked in all groups at 1-3 dpi. Nevertheless, doxycycline 25 mg/kg and omadacycline 10 mg/kg treatment groups showed significantly reduced clinical severity at 3 dpi compared to infection control. In all groups, clinical scores returned to baseline at 15 dpi. In addition, there was a significant reduction in body weight observed at 1, 3, 5, 7, 9, and 21 dpi compared to day 0, prior to wound infection, for all groups (Fig. 3B). Furthermore, a significant difference in body weight was found between the doxycycline 25 mg/kg group and the omadacycline 15 mg/kg group at 7 dpi, with the weight of the doxycycline-treated animals being slightly higher.

#### Bacterial clearance in tissues

**Fig 4:**
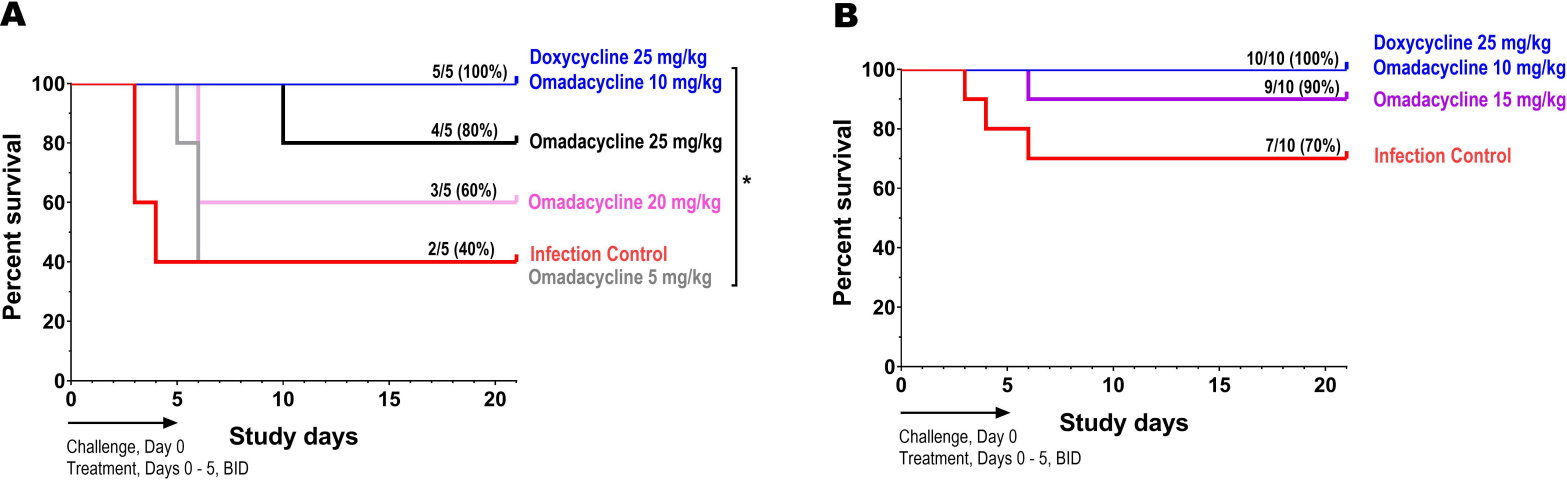
Survival of *A. baumannii*-infected mice in a chronic dorsal wound model after treatment with either doxycycline or omadacycline. Drugs were administered as doxycycline 25 mg/kg, or omadacycline 5, 10, 15, 20, and 25 mg/kg via IP injection on days 0–5. Infection control mice were administered PBS. Survival curves are presented for cohort 1 (**A**) and cohort 2 (**B**). *P*-value (log-rank test) is shown where survival was significantly different relative to the infection control (*, *P* < 0.05).

In cohort 1, it is difficult to compare the amount of bacteria present in wounds among the groups at the specific timepoint because most animal groups became moribund at different days before 7 dpi (Fig. 4A; Fig. S1); however, treatment groups had lower bacterial concentrations in the wound bed compared to the infection control group at variou time points depending on the onset of moribundity. *A. baumannii* was not detected in the wounds of mice treated with doxycycline 25 mg/kg and omadacycline 10 mg/kg and 20 mg/kg at 21 dpi (Fig. S1) but was detected in wounds of one mouse each from the infection control group, and the omadacycline (5 and 25 mg/kg) treatment groups at 21 dpi (Fig. S1). *A. baumannii* was detected in the spleens, livers, and lungs of all treatment groups at 3–10 dpi, indicating systemic spread of infection ,but was not observed at 21 dpi in any group (Fig. S1).

In cohort 1, the wounds of mice treated with doxycycline and all doses of omadacycline showed gradual healing, while the wounds of infection control mice were still open with scabs at the end of the study (day 21). Although all mice in the omadacycline treatment groups showed slightly delayed wound healing compared to the doxycycline treatment group, wound closure was substantially improved when compared to the infection control (Fig. S2). Among the different doses of omadacycline that were evaluated, 10 mg/kg and 20 mg/kg treatments resulted in complete clearing of *A. baumannii* infection in wounds, spleens, livers, and lungs at 21 dpi.

**Fig 5:**
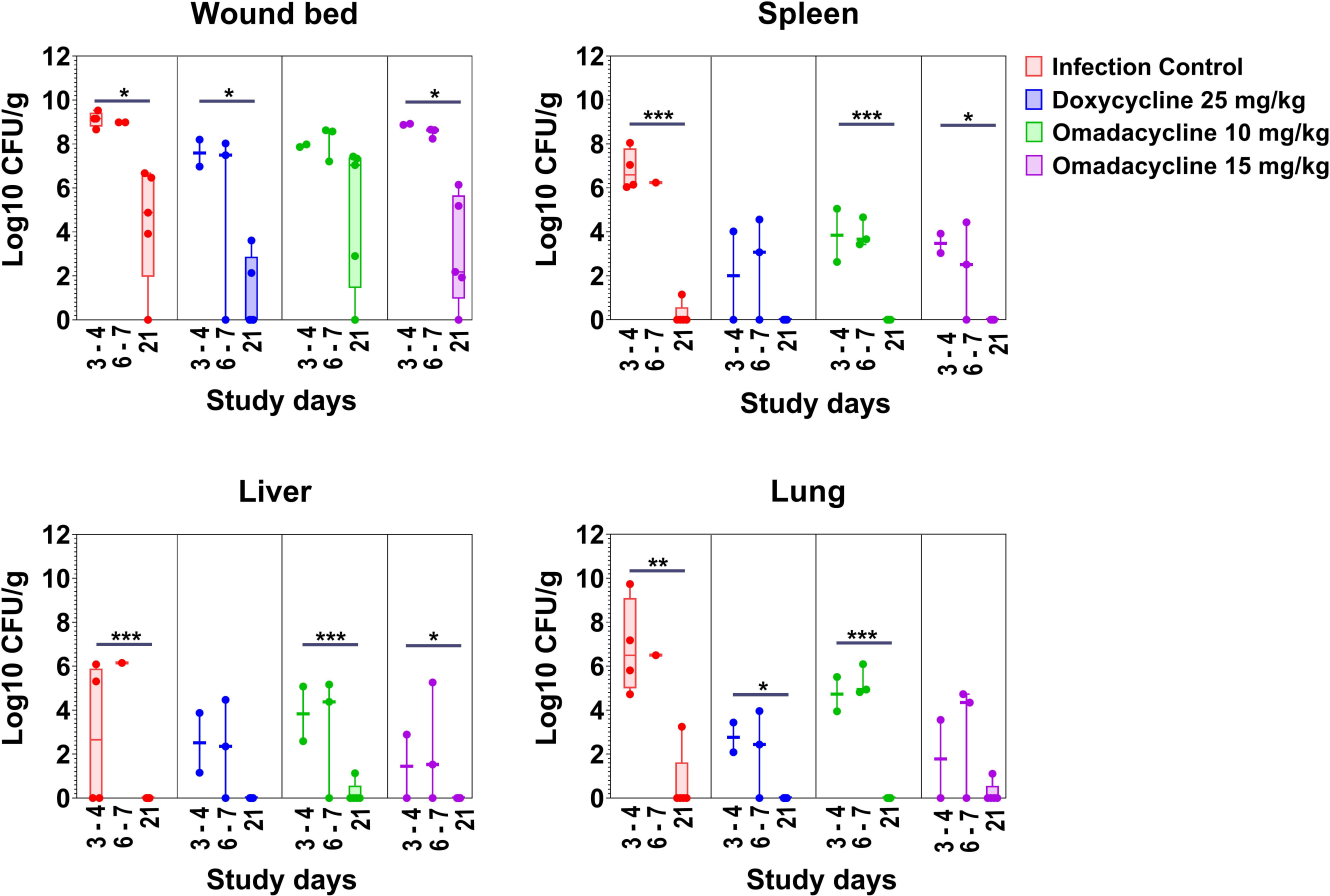
Cohort 2: Bacterial burden in wound beds, spleens, livers, and lungs of *A. baumannii*-infected mice treated with either doxycycline or omadacycline. Animals were treated with doxycycline 25 mg/kg, or omadacycline 10 and 15 mg/kg via IP injection on days 0–5. Infection control mice were administered PBS. The Box-and-Whisker plot shows the medians and interquartile ranges with 95% CI. Each dot represents data from each mouse. P-values are shown as significant differences relative to days 3–4 within the same group one-way ANOVA followed by Dunnett’s multiple comparisons test; *, *P* < 0.05; **, *P* < 0.01; ***, *P* < 0.001.

In cohort 2, the bacterial burden of wound beds and systemic organs (spleen, liver, and lung) was determined throughout the study to evaluate the efficacy of omadacycline and doxycycline compared to infection control. As shown in Fig. 5, the bacterial burden for all tissues gradually decreased over time for all treatment groups. At 21 dpi, treatment with omadacycline 10 mg/kg demonstrated bacterial clearance in the spleen (*n* = 5/5), liver (*n* = 4/5), and lungs (*n* = 5/5), and treatment with omadacycline 15 mg/kg showed similar clearance of bacteria in the spleen (*n* = 5/5), liver (*n* = 5/5), and lungs (*n* = 4/5). Treatment of doxycycline 25 mg/kg demonstrated complete bacterial clearance in the spleen (*n* = 5/5), liver (*n* = 5/5), and lungs (*n* = 5/5), while the infection control demonstrated bacterial clearance in the spleen (*n* = 4/5), liver (*n* = 5/5), and lungs (*n* = 4/5) at 21 dpi.

In wound beds of cohort 2, the average bacterial count (mean log10 CFU/g) at 21 dpi for the surviving animals was significantly lower compared to 3–4 dpi the infection control group (4.39 vs 9.13, *P* = 0.012), doxycycline 25 mg/kg (1.15 vs 7.59, *P* = 0.043), and omadacycline 15 mg/kg (3.09 vs 8.90, *P* = 0.014). In spleens, *A. baumannii* was not detected at 21 dpi for any treatment group, while a low bacterial burden (0.23 log10 CFU/g) was present in the spleen of one of two surviving mice in the infection control group. The average CFUs in spleens at 21 dpi were significantly different than at 3–4 dpi in all treatment groups except the doxycycline treatment group (below the limit of detection (BLD) vs 2 log10 CFU/g). The mean reduction of bacteria (log10 CFU/g) in spleens at 21 dpi was greater than that at 3–4 dpi for omadacycline 10 mg/kg (BLD vs 3.84, *P* = 0.0007), omadacycline 15 mg/kg (BLD vs 3.48, *P* = 0.019), and the surviving infection control group (0.23 vs 6.82, *P* < 0.0001) (Fig. 4). Similarly, CFU count in the lungs was statistically reduced over time (21 dpi vs 3–4 dpi) for the surviving infection control group (0.65 vs 6.86, *P* = 0.003), doxycycline (BLD vs 2.76, *P* = 0.037), and omadacycline 10 mg/kg (BLD vs 4.73, *P* < 0.0001). In the liver, there was no presence of bacteria at 21 dpi in any group (BLD) except a low burden (1.1 log10 CFU/g) in one of five mice in the omadacycline 10 mg/kg group. *A. baumannii* persisted in the lungs at 21 dpi in 1/5 mice of the infection control group and 1/5 mice of the omadacycline 15 mg/kg group, but bacterial counts in the infection control group were substantially higher than the omadacycline 15 mg/kg-treated group (3.25 vs 1.11 CFU/g).

**Fig 6:**
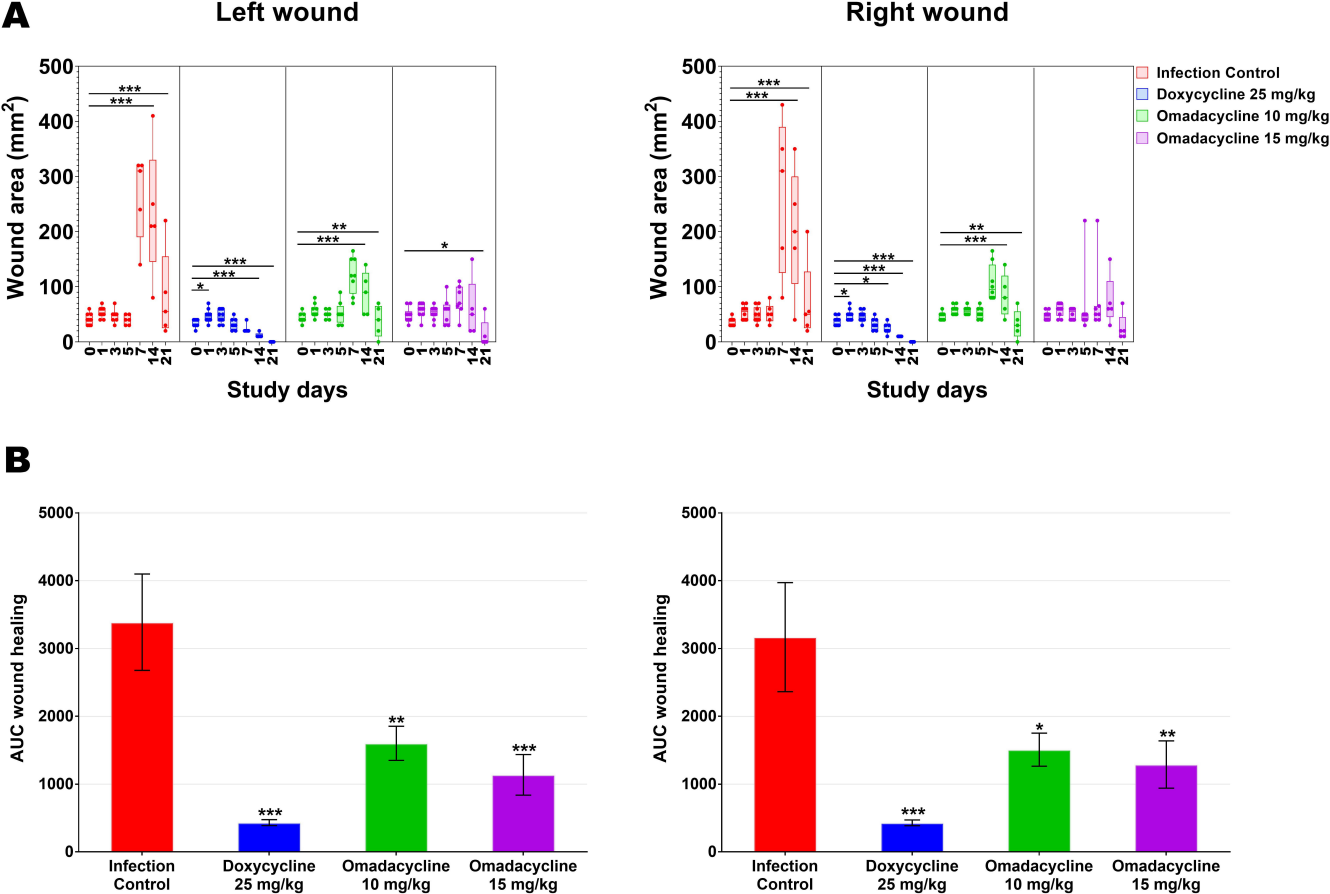
Cohort 2: Time courses of wound area size of *A. baumannii*-infected mice treated with either doxycycline or omadacycline. (**A**) The Box-and-Whisker plot shows the medians and interquartile ranges with 95% CI. Each dot represents data from each mouse. *P-*values are shown as significant differences relative to day 0 within the same group. (**B**) Graphs show mean areas under the wound area-versus-time curves in the two-dimensional coordinate system in panel **A**, Representing the overall wound healing. Bars show mean ± standard error of the mean. *P*-values are shown as significant differences between drug treatment groups and infection control. One-way ANOVA followed by Dunnett’s multiple comparisons test; *, *P* < 0.05; **, *P* < 0.01; ***, *P* < 0.001. AUC, area under the curve.

**Fig 7:**
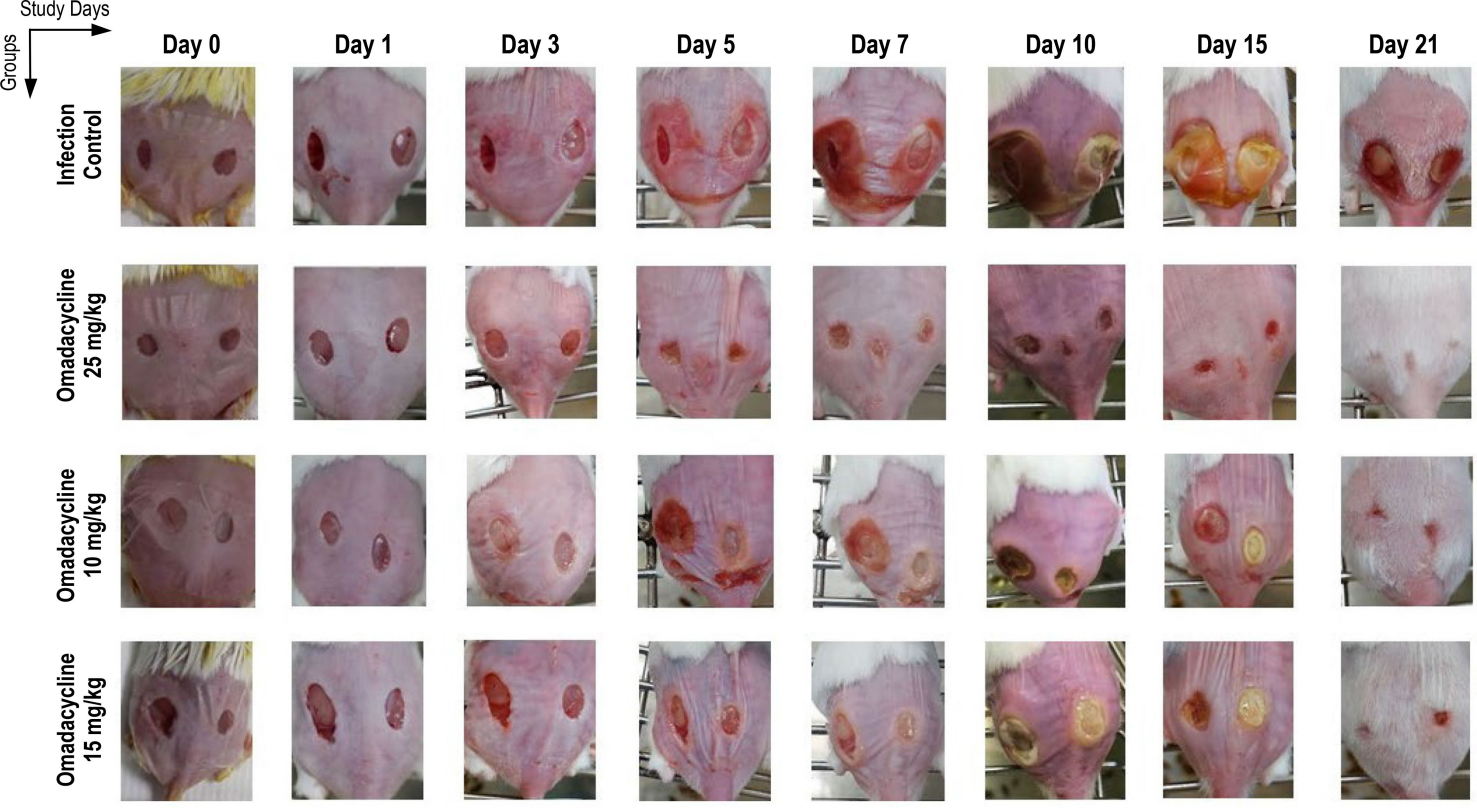
Cohort 2: Time courses of wound photographs for *A. baumannii*-infected mice treated with either doxycycline or omadacycline. In each group, images represent one mouse throughout the study period.

As depicted in Fig. 6 and 7, treatment with omadacycline 10 and 15 mg/kg in cohort 2 promoted skin wound healing, although the wound closure was slightly delayed compared to that of the doxycycline 25 mg/kg group. The average wound areas in the omadacycline groups (10 and 15 mg/kg) were significantly decreased starting at 14 dpi and/or 21 dpi compared to those on day 0, while in the doxycycline group, wound areas were significantly reduced starting on dpi 7 and continued on dpi 14 and/or 21 (Fig. 6A). In addition, the open wounds of doxycycline-treated mice started healing on days 5 and 7, whereas the open wounds in the omadacycline 10 and 15 mg/kg treatment groups started healing on days 14 and 21 (Fig. 6A and 7). By area under the curve (AUC) analysis of mean wound areas, the overall wound areas in the omadacycline 10 and 15 mg/kg groups were significantly decreased compared to infection control (Fig. 6B). The improved wound closure correlated with increased body weight and decreased clinical sign severity.

### Host and microbial responses to treatment with omadacycline in a dorsal wound murine model (cohort 2)

#### Inflammatory responses

**Fig 8:**
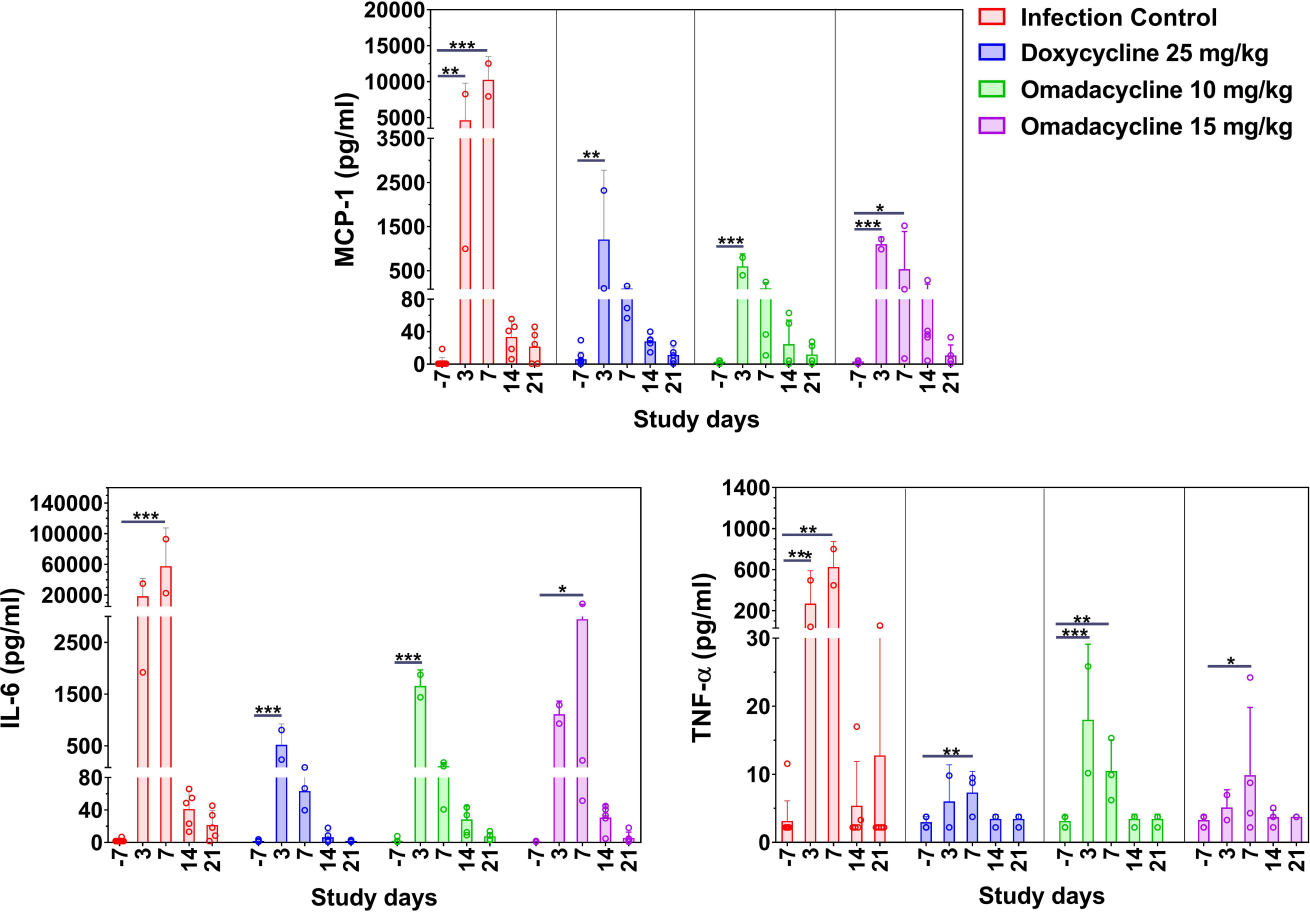
Cohort 2: Pro-inflammatory cytokines in plasma from the *A. baumannii*-infected mice treated with either doxycycline or omadacycline. Bar shows the mean ± standard deviation, and each dot represents data from each mouse. *P*-values are shown significant differences relative to day (−7) as a baseline within the same group. One-way ANOVA followed by Dunnett’s multiple comparisons test; *, *P* < 0.05; **, *P* < 0.01; ***, *P* < 0.001.

In cohort 2, the levels of systemic pro-inflammatory cytokine biomarkers IL-6 and TNF-α, as well as the chemokine MCP-1, in plasma samples of mice infected with *A. baumannii* in the dorsal wound model were evaluated. In all treatment groups, a significant increase in inflammatory cytokine levels at 3 and/or 7 dpi compared to day 7 (i.e., prior to infection) was observed (Fig. 8). The levels of IL-6, TNF-α, and MCP-1 at 7 dpi in the doxycycline 25 mg/kg and omadacycline 10 and 15 mg/kg treatment groups were significantly lower than the infection control group. Further, the gradual reduction in inflammatory cytokines observed over time was consistent with wound healing and showed no significant differences between the doxycycline and omadacycline treatment groups on 14 and 21 dpi.

#### Histopathologic evaluation of wounds

**Fig 9:**
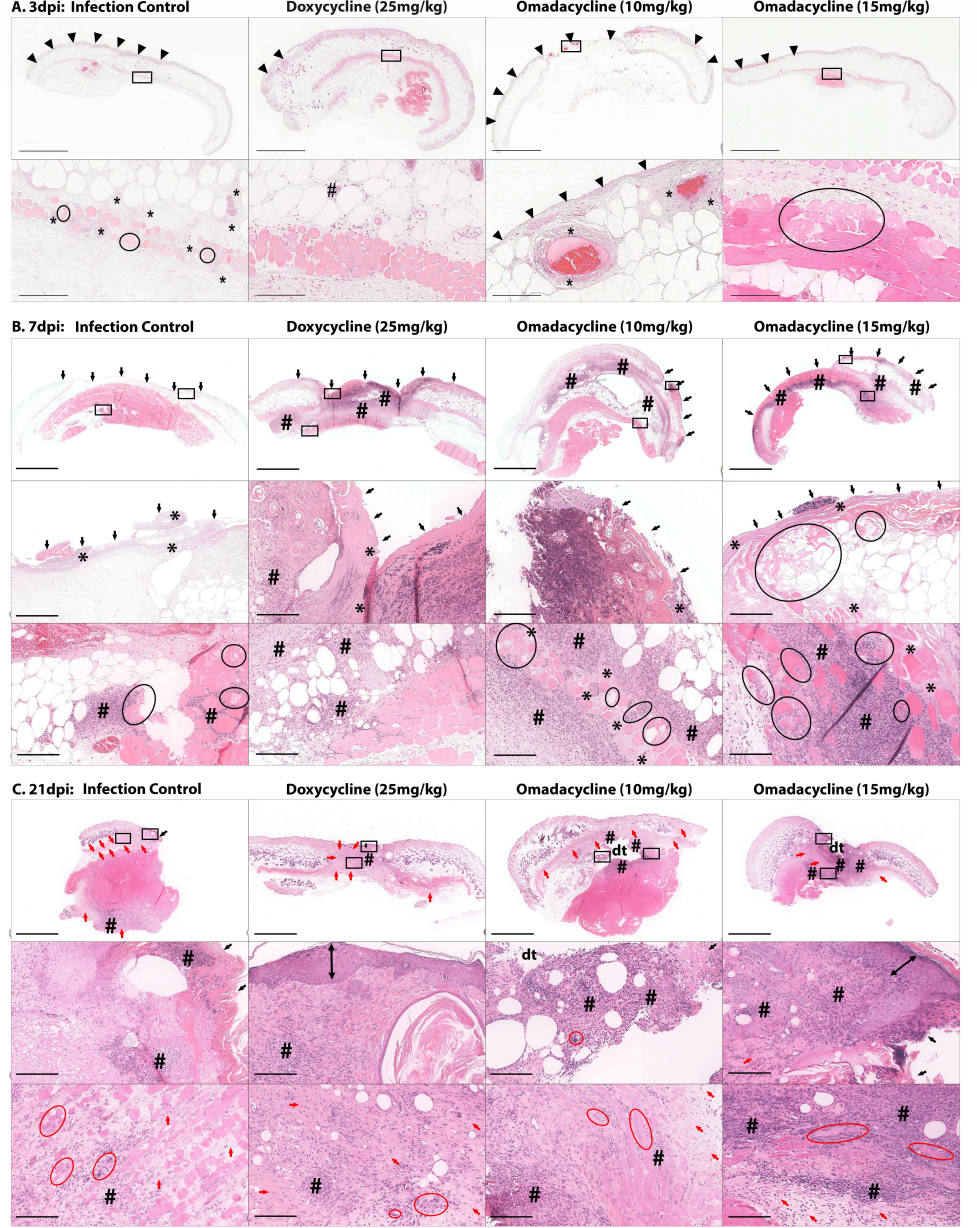
Cohort 2: Combined representative H&E images from the wound beds of *A. baumannii*-infected mice. Infection control, doxycycline 25, omadacycline 10, and 15 mg/kg-treated mice at 3–4 dpi (**A**), 6–7 dpi (**B**) ,and 21 dpi (**C**). Each column shows representative lesions from a single animal, with black tooling lines denoting individual images. Lower panels are areas denoted by the black box in the upper panel. Black arrow-heads = epidermal discontinuity/loss; asterisks = colonies of cocci; # = mixed inflammation; black arrows = epidermal ulceration with serocellular crusts; black circles = skeletal muscle necrosis; double-headed black arrows = epidermal hyperplasia; red arrows = granulation tissue progressing to mature fibrosis; red circles = skeletal muscle regeneration; dt = draining tract. Scale bar = 2 mm (subgross images, top row, **A–C**); scale bar = 200 µm (higher magnification images, middle and bottom rows, **B–C**).

**Fig 10:**
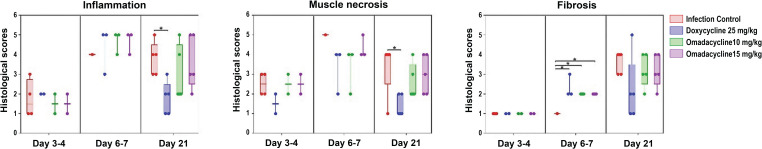
Cohort 2: Histopathological scores of wound beds of *A. baumannii*-infected mice. Infection control, doxycycline 25 mg/kg, omadacycline 10 mg/kg, and 15 mg/kg-treated mice at 3–4 dpi, 6–7 dpi, and 21 dpi. The box-and-whisker plot shows the medians and interquartile ranges with 95% CI. Each circle represents one mouse. One-way ANOVA followed by Dunnett’s multiple comparisons test; *, *P* < 005.

H&E-stained slides containing wound biopsies were examined microscopically to assess the presence of bacteria, inflammation, and indications of wound healing, that is, extent of re-epithelization and amount of granulation tissue and/or fibrosis present within the wound. Microscopic images from the wound beds of the infection control, doxycycline 25 mg/kg, omadacycline 10 mg/kg, and omadacycline 15 mg/kg treatment groups are shown in Fig. 9. At 3–4 dpi, wounds typically exhibited small foci of epidermal discontinuity and loss associated with large colonies of cocci, which extended into the deep subcutis and panniculus; however, inflammation was minimal at this time point (Fig. 9A). Overall lesion severity, characterized by epidermal ulceration with serocellular crust formation, large colonies of cocci, extensive, full-thickness inflammation, and rhabdomyocyte (skeletal muscle) necrosis, peaked at 6–7 dpi in all treatment groups (Fig. 9B). At the end of the study, bacterial colonies were not evident in any examined tissue section, and all examined wounds demonstrated some evidence of tissue repair, including an intact, often hyperplastic epidermis, mild inflammation, variably mature fibrous connective tissue, and/or skeletal muscle regeneration (Fig. 9C). Animals with residual ulceration at 21 dpi included four infection control mice and two omadacycline 15 mg/kg-treated mice. The two latter mice, as well as one omadacycline 10 mg/kg-treated mouse, also exhibited evidence of draining tracts. There was no significant difference in histology severity score between the treatment groups at 3–4 dpi; however, at 6–7 dpi, wound beds from both of the omadacycline-treated groups and the doxycycline-treated group exhibited significantly more fibrosis than the infection control group, and at 21 dpi, there was significantly decreased inflammation and rhabdomyocyte necrosis in the doxycycline-treated group when compared to the infection control group (Fig. 10).

#### Biofilm disruption

**Fig 11:**
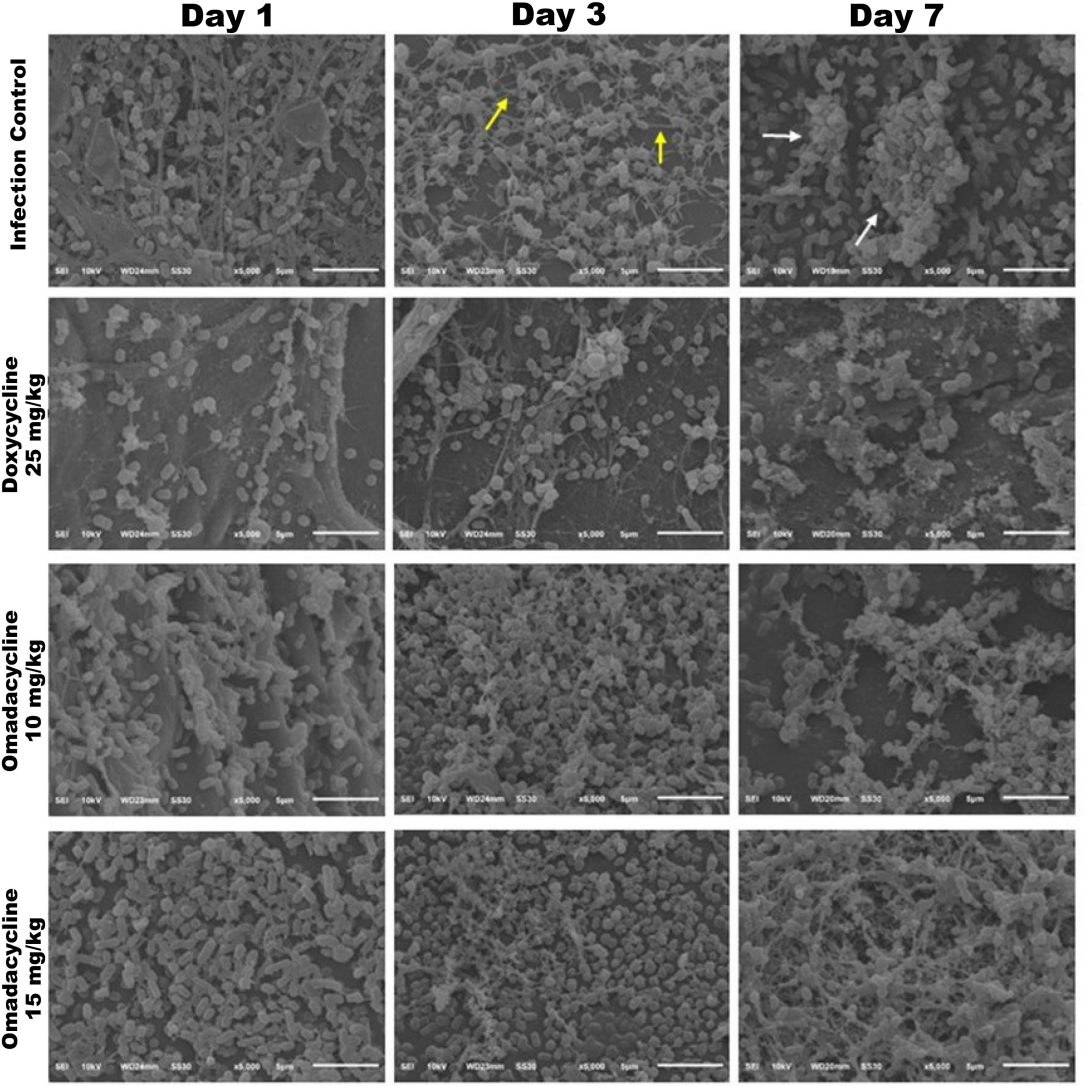
Cohort 2: SEM of TegadermTM dressings covering the *A. baumannii*-infected wounds treated with either doxycycline or omadacycline. Tegaderm dressings were collected at 1, 3, and 7 dpi. Yellow arrows indicate long cell extensions with a complex exopolysaccharide structure on 3 dpi; white arrows indicate mature biofilm on 7 dpi in the infection control group (20, 22). Images represent data from one mouse. Images show magnification of 5,000; Bars - 5 µm.

Biofilm disruption due to antibiotic treatment was observed by SEM analysis on the Tegaderm wound dressings collected at 1, 3, and 7 dpi. In the infection control group at 3 dpi (Fig. 11), bacteria-produced extracellular matrix and fibroblasts on the wound surface were observed. The formation of mature biofilm was visualized at 7 dpi. SEM imaging at 3 dpi showed reduced exopolysaccharide production on the wound dressing of doxycycline-treated mice compared to the infection control. In addition, the omadacycline-treated groups were observed to have a reduction in the complex exopolysaccharide structure compared to the infection control group. Lastly, and in contrast to the infection control group, mature biofilms on the wound dressings at 7 dpi were not seen in any treatment groups (Fig. 11).

#### Microbial community of wounds

**Fig 12:**
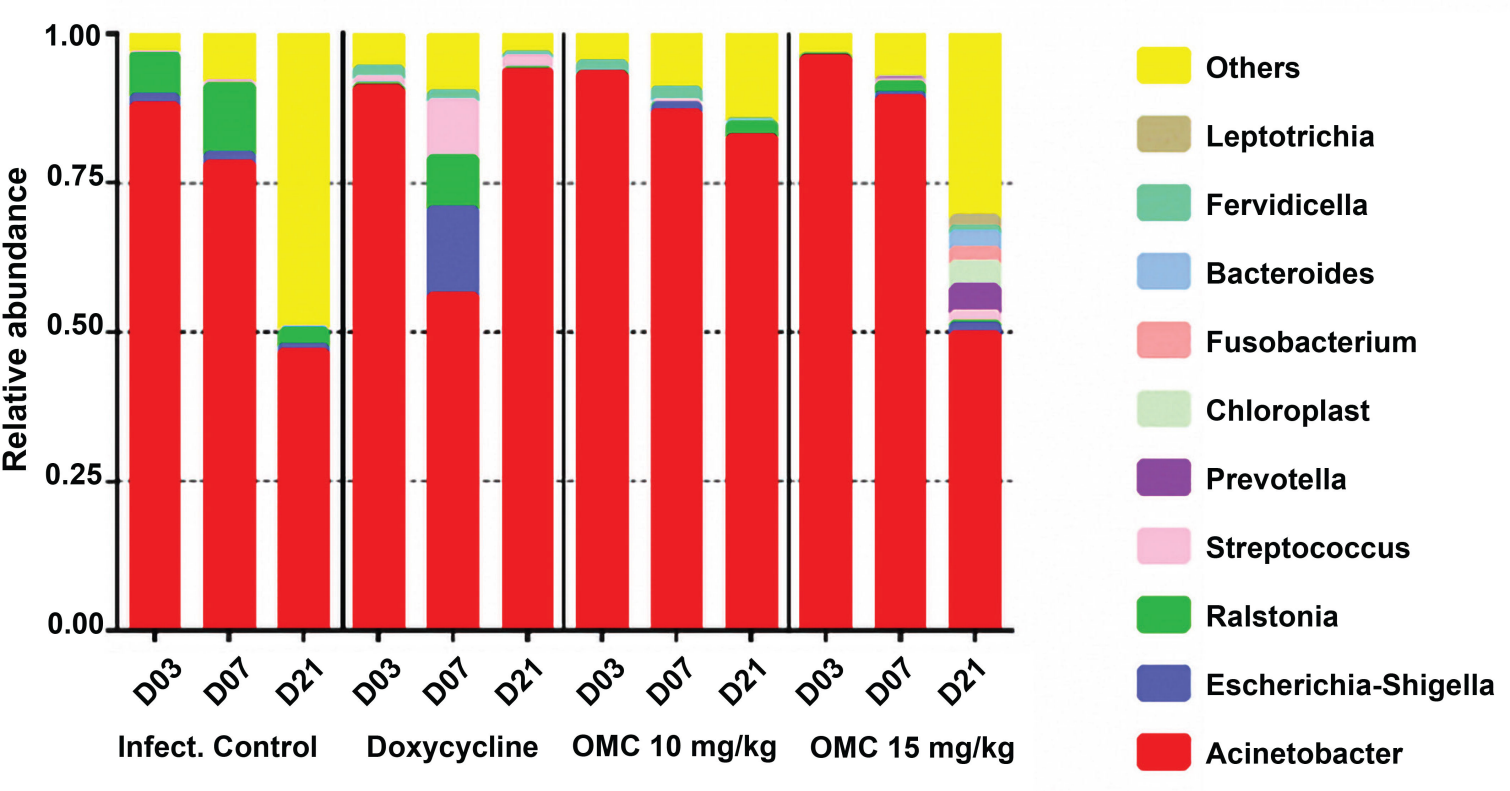
Relative abundance of the microbial community at wound sites of mice infected with *A. baumannii* and treated with either doxycycline or omadacycline. D03: *N* = 2 for each group; D6: *N* = 1 for infection control group; D07: *N* = 3 doxycycline and omadacycline, each; D21: *N* = 2 for infection control group, *N* = 1 for doxycycline, and *N* = 2 for omadacycline. The species listed represent the top 10 taxa identified. The top 30 taxa included *Thermoanaerobacterium*, *Thiomonas*, *Neisseria*, *Arthrobacter*, *Staphylococcus*, *Veillonella*, *Lactobacillus*, *Haemophilus*, *Clostridium sensu stricto 7*, *Pseudomonas*, *Faecalibacterium*, *Ruminococcaceae*, *Photobacterium*, *Corynebacterium*, *Rhodanobacter*, *Methylobacterium-Methylorubrum*, *Candidatus Aquiluna*, *Alloprevotella*, Mitochondria (*Rickettsiales* family), and *Incertae sedis*.

**Fig 13:**
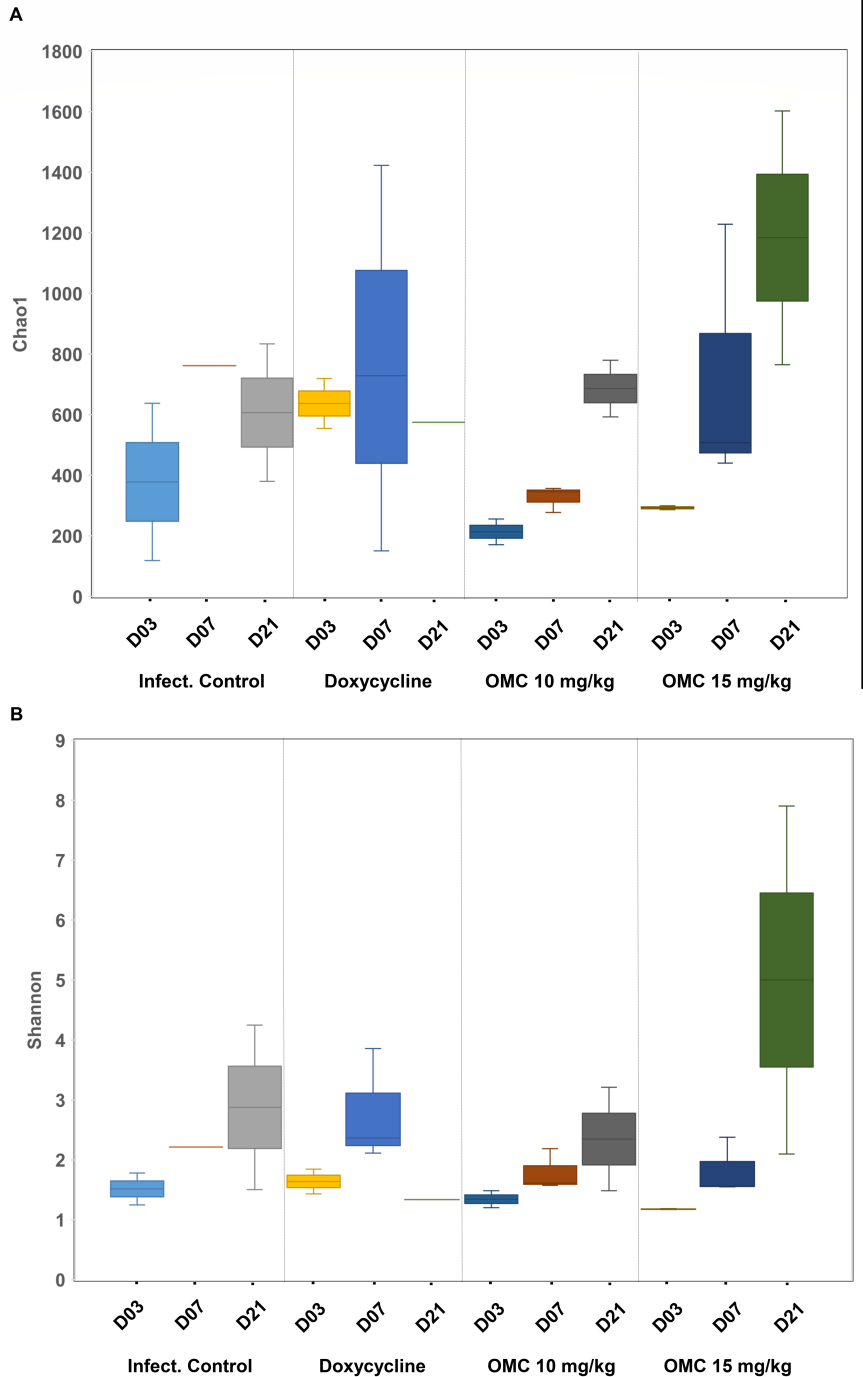
Cohort 2: Changes in alpha-diversity over the antibiotic recovery period for wound beds in *A. baumannii*-infected mice treated with doxycycline or omadacycline. (**A**) Chao1 group and (**B**) Shannon methods.

**Fig 14:**
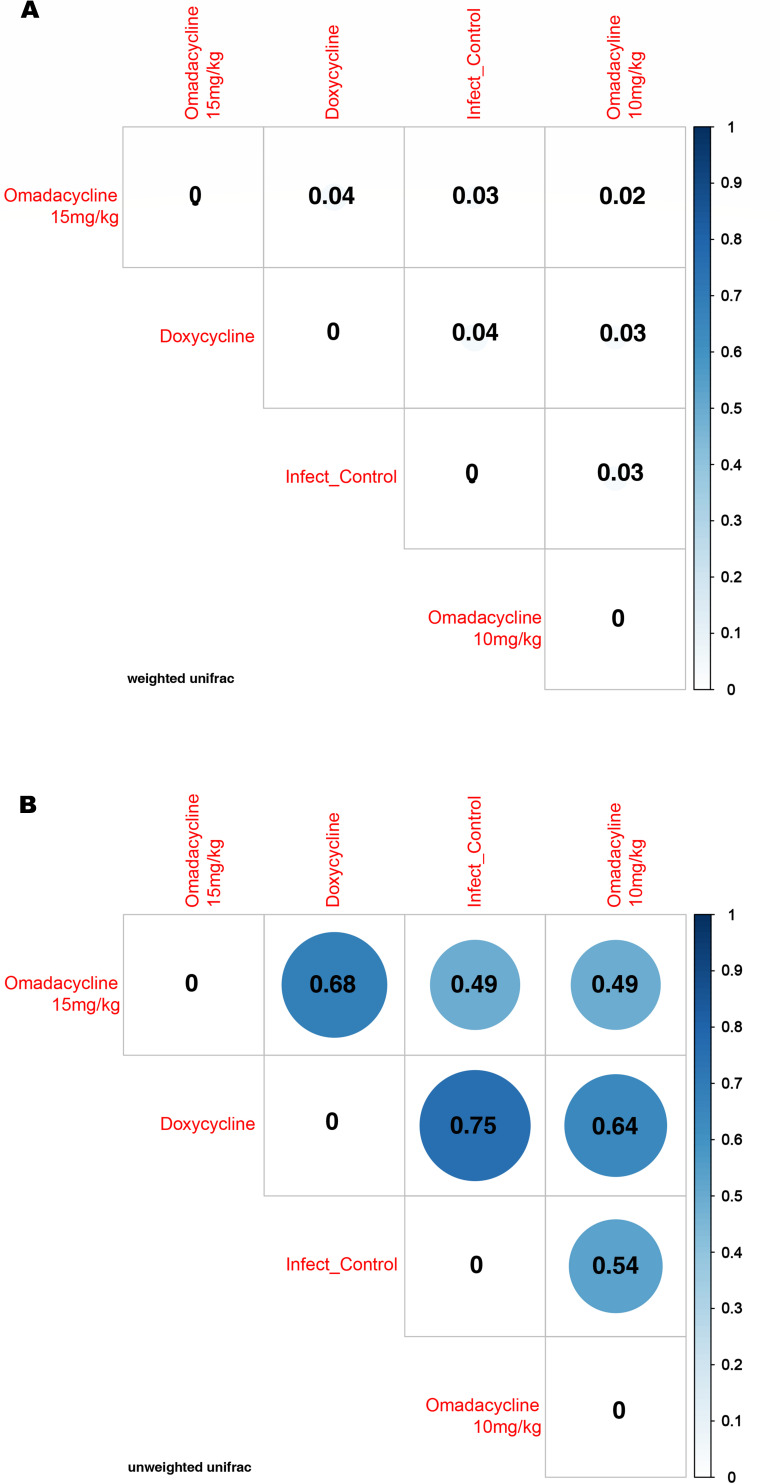
Beta-diversity heatmap comparing taxa found in wound beds of *A. baumannii*-infected mice among the doxycycline and omadacycline treatment groups. (**A**) Weighted UniFrac and (**B**) unweighted UniFrac.

The microbial community in the wound beds was evaluated by 16S sequencing at 3, 7, and 21 dpi and compared across groups. *Acinetobacter* spp. remained at a relatively high abundance throughout the experiment with a notable decrease at 21 dpi for infection control and omadacycline 15 mg/kg treatment groups (Fig. 12). The relative abundance of *Acinetobacter* spp. dropped to ~50% in both the infection control group and the omadacycline 15 mg/kg group, but the microbial diversity in the omadacycline 15 mg/kg treatment group was higher (Fig. 12 and 13). The abundance of *Acinetobacter* spp. dropped to ~50% at 7 dpi in the doxycycline 25 mg/kg treatment group, concurrent with the expansion of *Escherichia*, *Shigella*, and *Streptococcus*, and then increased back to ~90% at 21 dpi. This increase does not correlate with the reported CFU for *A. baumannii* (Fig. 5), which could be due to the utilization of different wound beds (left and right) from only one mouse for both analyses. Both omadacycline treatment groups demonstrated relative reduction of *A. baumannii* abundance over time with an increase in alpha diversity over time as well (Fig. 13). The omadacycline 10 mg/kg group had a similar increase in diversity to the infection control group, while the omadacycline 15 mg/kg group had a much greater increase in diversity over time compared to the infection control group, and the doxycycline 25 mg/kg group did not demonstrate an increase in diversity at the wound site. The microbial abundance was relatively similar among all of the treatment groups, but the doxycycline treatment group was the least similar to the others in terms of diversity (Fig. 14).

With these two mouse models, the neutropenic thigh mouse model, considered to be a gold standard for efficacy evaluation of anti-infective agents, provided the first *in vivo* model to explore the effects of omadacycline on the reduction of bacteria at the initial stage for dose optimization to be further evaluated in chronic wound infection model that presents the complex environment of persistence, non-healing wound, and biofilm formation for drug development.

## DISCUSSION

Omadacycline demonstrated potent *in vitro* activity against a geographically, genetically, and phenotypically diverse panel of MDR clinical isolates obtained through MHS surveillance efforts, with activity similar to that of tigecycline, while doxycycline was less active *in vitro*. The results from our *in vitro* study align with previously published data for omadacycline, where an omadacycline MIC_50/90_ of 0.5/4 µg/mL was reported for *A. baumannii* isolates (*N* = 76) collected from across the United States in a 2019 surveillance study (23). Omadacycline showed the highest MIC value at 8 µg/mL against two distinct bacterial isolates. Sequencing data revealed no tetracycline resistance genes, suggesting omadacycline resistance wasn’t mediated by typical tetracycline mechanisms. However, both isolates harbored β-lactamase genes and resistance genes for sulfonamides and aminoglycosides, indicating broader resistance (data not shown). Antibiotics from these classes weren’t evaluated in this study. These findings highlight complex resistance mechanisms and the importance of evaluating a comprehensive range of antibiotic classes when investigating novel compounds such as omadacycline (15).

In a mouse neutropenic thigh infection model, omadacycline treatment (10 and 25 mg/kg) showed a dose-dependent reduction in *A. baumannii* bacterial load and demonstrated efficacy similar to tigecycline (10 and 25 mg/kg). These results are in line with previous reports utilizing a similar model, where omadacycline was found to be efficacious in a dose-dependent manner in murine neutropenic thigh and neutropenic pneumonia infection models against *S. aureus* (24, 25). Based on previous PK studies in mice, the omadacycline and doxycycline doses evaluated in this study are expected to result in exposures similar to that of humans when administered the respective US FDA-approved dosing regimens (26, 27). However, while the doses of tigecycline evaluated in the mouse model were chosen as a positive control, the exposures in mice are expected to be higher than that of the human administered FDA-approved dose (28).

In the dorsal wound mouse model of chronic *A. baumannii* infection, treatment with either omadacycline (10 mg/kg) or doxycycline (25 mg/kg) accelerated wound healing over time. For wound closure, doxycycline 25 mg/kg-treated wounds appeared grossly healed by 15 dpi, while omadacycline (10 and 15 mg/kg)-treated wounds exhibited a slight delay compared to doxycycline. Wound healing was similar for the omadacycline 10 and 15 mg/kg groups across cohorts 1 and 2. Mice from all treatment groups (doxycycline and omadacycline) exhibited a relatively normal progression of tissue repair as determined by histology; however, lesion severity and the degree of healing varied based on day post-inoculation and treatment group. At 3–4 dpi, there was no significant difference in any histology severity score between any treatment group, which is an expected finding at the early time point. Overall lesion severity peaked at 6–7 dpi, and at this time point, both omadacycline-treated groups, as well as the doxycycline-treated group, exhibited significantly higher microscopic fibrosis scores (a tissue repair indicator) than the infection control group. By 21 dpi, only doxycycline treatment was associated with a significant decrease in inflammation and notable reduction in skeletal muscle necrosis when compared to the infection control group, while omadacycline treatment at either dose did not exert a statistically significant effect. This could simply be a function of group size, individual variability in the immune response over the course of *A. baumannii* infection and/or perhaps relatively decreased systemic/wound bed omadacycline persistence (vs doxycycline).

We demonstrated that *A. baumannii* bacterial burden in the wound bed decreased from days 3/4 to day 21 in all groups. *A. baumannii* was completely eradicated after treatment with doxycycline at 25 mg/kg in 3/5 mice and omadacycline at the middle doses (10, 20 mg/kg) in 4/5 mice, but not at the lowest (5 mg/kg) or highest dose (25 mg/kg) tested (data not shown). The bacterial burden in the wound bed was highly variable in all groups, which may reflect variability in the immune response over the course of infection. These results imply that not only is there a potential dose effect, which explains lack of full clearance with the lower dose but also possibly a lack of tolerance in this model at the highest dose tested.

The ability of *A. baumannii* to persist in wound beds through day 21 may be due to the establishment of bacterial biofilms. At 7 dpi, omadacycline treatment at both 10 and 15 mg/kg showed an effective reduction of *A. baumannii* biofilms in the wound beds compared to infection control mice, where robust biofilms were observed. Of note, it has been previously reported that various ecological niches, that is, skin and soft tissue infections, wound dressing, and abiotic surfaces, can enhance biofilm formation of *A. baumannii* (29). Biofilm formation could therefore lead to limited access of drugs to the specific target as well as reduced efficacy of host immune responses (30–32). It has been shown that biofilms in wounds are one of the major factors influencing the failure of antibiotic treatment (32). Further, the prolonged infection or delayed clearance of *A. baumannii* in wounds is prone to cause re-infection. Therefore, it is possible that a higher antibiotic dose, a longer antibiotic course, or a topical treatment may improve the clearance of *A. baumannii* bacterial skin infections. This highlights the importance of combined systemic and local treatment to completely eradicate *A. baumannii* infection and prevent re-infection.

The 16S microbial sequencing analysis of wound beds revealed that the abundance of *A. baumannii* DNA decreased over time across all groups except the doxycycline group by day 21. The wound bed of the infection control group was comprised mostly of *Acinetobacter* and *E. coli*. The abundance of *Acinetobacter* spp. in the omadacycline 15 mg/kg group decreased over time to the same level as the infection control group but had greater microbial diversity than the infection control group at 21 dpi. The omadacycline 10 mg/kg-treated group had a smaller reduction in *A. baumannii* at 21 dpi when compared to the 15 mg/kg group, but the level of microbial diversity at 21 dpi was similar to the infection control group. In contrast, treatment with doxycycline led to an expansion of *E. coli/Shigella* and *Streptococcus* at 7 dpi. The diversity of the wound microbiome of the doxycycline-treated group did not increase in parallel with wound healing and had the lowest bacterial burden at day 21. However, the doxycycline-treated group also had the highest abundance of *A. baumannii* DNA at 21 dpi, suggesting that the levels of *A. baumannii* as determined by 16S are driven, at least in part, by dead bacteria. These data suggest that the recovery of the microbial community after a wound infection when treated by omadacycline resembles that of an untreated wound but occurs at a more rapid rate and that treatment with omadacycline promotes a more diverse microbiome on recovery. The sample size of this study was small for each group, and only the top 30 species were identified; therefore, future studies would be needed to fully understand the microbiome in the wound bed, but this was beyond the scope of this project. These results suggest that doxycycline has a more detrimental effect on the skin microbiome and could lead to outgrowth of other pathogenic bacteria. The data here are in line with previous data showing that omadacycline is less detrimental to the microbiome compared to other antibiotics and promotes the recovery of microbiome diversity (33).

We also demonstrated that *A. baumannii* present in topically infected wounds could disseminate into internal organs to cause a systemic infection as evidenced by the presence of bacteria in the spleen, liver, and lungs of mice at 7 dpi. Treatment with either omadacycline (10 mg/kg) or doxycycline (25 mg/kg) increased survival to 100% for mice with wounds infected by *A. baumannii*. The production of pro-inflammatory cytokines, IL-6, MCP-1, and TNF-α, was substantially elevated at 3 or 7 dpi in the infection control group relative to the treatment groups. These results suggest that wounds infected with *A. baumannii* led to a high degree of systemic inflammation concordant with systemic spread of the bacteria. Treatment with omadacycline or doxycycline reduced the severity of inflammatory responses in conjunction with improved wound healing, bacterial clearance, and improved survival. Tetracycline class drugs are known to have anti-inflammatory effects, and this is supported by the data presented here (34). At day 21, bacterial burden in the spleen was reduced to BLD in all treatment groups and in all but one animal in the infection control group. A single omadacycline 10 and 15 mg/kg-treated mouse had a very low bacterial burden in the liver and lung, respectively, at the end of the study despite bacteria still being present in the wound bed. The severity of clinical signs of mice in all treatment groups, including the infection control group, peaked at 3 or 5 dpi, but the omadacycline- and doxycycline-treated mice had less severe clinical signs than the infection control mice. All mice in the infection control and treatment groups resumed normal behavioral signs at 15 dpi, except for one doxycycline-treated mouse that had a moderately rough coat. It is likely that the rapid clearance of systemic infection was related to the quick recovery of clinical signs and immune response.

In conclusion, omadacycline demonstrated potent *in vitro* activity against geographically, genetically, and phenotypically diverse *A. baumannii* clinical isolates. In addition, this is the first report, to our knowledge, to evaluate and demonstrate *in vivo* efficacy of omadacycline against *A. baumannii* using two different mouse models, that is, the neutropenic thigh infection model and chronic wound infection model. Together, the data from these *in vitro* and *in vivo* studies support further investigation of omadacycline as a potential treatment for infections caused by *A. baumannii*.

## SUPPLEMENTAL MATERIAL

Supplemental material aac.00932-25-s0001.docx Table S1; Fig. S1 and S2.

ASM does not own the copyrights to Supplemental Material that may be linked to, or accessed through, an article. The authors have granted ASM a non-exclusive, world-wide license to publish the Supplemental Material files. Please contact the corresponding author directly for reuse.
