# Supplementary material for: Preclinical in vitro and in vivo evaluation of omadacycline against multidrug-resistant Acinetobacter baumannii
Source: Antimicrob Agents Chemother. 2025 Oct 30;69(12):e00932-25. doi: 10.1128/aac.00932-25 (PMC12691642; doi:10.1128/aac.00932-25)
Supplement: Supplemental material — Table S1; Fig. S1 and S2. [file aac.00932-25-s0001.docx]

**Supplemental material**

**Table S1: *In vitro* activity of omadacycline and comparator antibiotics against *A. baumannii* clinical isolates (N=100).**

| **MRSN ID** | **MLST** | **Omadacycline MIC** | | **Doxycycline MIC** | | **Tigecycline MIC** | | **Levofloxacin MIC** | |
| --- | --- | --- | --- | --- | --- | --- | --- | --- | --- |
|  |  | A | B | A | B | A | B | A | B |
| 334 | 2 | 1 | 1 | ≤ 0.25 | ≤ 0.25 | 0.5 | 0.5 | 4 | 4 |
| 843 | 417 | 1 | 1 | 1 | 1 | 0.5 | 0.5 | 16 | 16 |
| 918 | 2 | 2 | 2 | 32 | 32 | 1 | 1 | 4 | 4 |
| 959 | 1 | 0.5 | 0.5 | ≤ 0.25 | ≤ 0.25 | ≤ 0.25 | ≤ 0.25 | 4 | 4 |
| 960 | 1 | 2 | 2 | 64 | 64 | 1 | 1 | 8 | 8 |
| 1171 | 2 | 1 | 1 | 8 | 8 | 0.5 | 0.5 | 2 | 4 |
| 1174 | 2 | 2 | 2 | 1 | 1 | 1 | 2 | 32 | 32 |
| 1183 | 267 | ≤ 0.25 | 0.5 | 32 | 32 | ≤ 0.25 | ≤ 0.25 | 8 | 8 |
| 1187 | 79 | 1 | 1 | ≤ 0.25 | ≤ 0.25 | 0.5 | 0.5 | 8 | 16 |
| 1196 | 108 | 1 | 1 | ≤ 0.25 | ≤ 0.25 | 0.5 | 0.5 | 8 | 4 |
| 1311 | 1 | 4 | 4 | 4 | 4 | 1 | 1 | 4 | 4 |
| 1551 | 10 | ≤ 0.25 | ≤ 0.25 | 4 | 4 | 0.5 | 0.5 | 0.5 | 0.5 |
| 2821 | 537 | ≤ 0.25 | ≤ 0.25 | 0.5 | 0.5 | ≤ 0.25 | ≤ 0.25 | ≤ 0.25 | ≤ 0.25 |
| 3360 | 499 | 2 | 2 | 0.5 | 0.5 | 1 | 1 | 8 | 8 |
| 3658 | 54 | ≤ 0.25 | ≤ 0.25 | ≤ 0.25 | ≤ 0.25 | ≤ 0.25 | ≤ 0.25 | ≤ 0.25 | ≤ 0.25 |
| 3692 | 32 | ≤ 0.25 | 0.5 | ≤ 0.25 | ≤ 0.25 | ≤ 0.25 | ≤ 0.25 | ≤ 0.25 | ≤ 0.25 |
| 3874 | 111 | 1 | 0.5 | ≤ 0.25 | ≤ 0.25 | 0.5 | 0.5 | 8 | 8 |
| 4484 | 195 | ≤ 0.25 | ≤ 0.25 | 2 | 2 | ≤ 0.25 | ≤ 0.25 | 4 | 4 |
| 4943 | 2 | 2 | 2 | 16 | 16 | 1 | 1 | 4 | 4 |
| 5969 | 239 | ≤ 0.25 | ≤ 0.25 | ≤ 0.25 | ≤ 0.25 | ≤ 0.25 | ≤ 0.25 | ≤ 0.25 | ≤ 0.25 |
| 6541 | 1 | 0.5 | 0.5 | ≤ 0.25 | 0.5 | 0.5 | 0.5 | 32 | 32 |
| 7067 | 81 | 8 | 8 | 0.5 | 0.5 | 2 | 2 | 8 | 8 |
| 7113 | 973 | ≤ 0.25 | ≤ 0.25 | ≤ 0.25 | ≤ 0.25 | ≤ 0.25 | ≤ 0.25 | ≤ 0.25 | ≤ 0.25 |
| 7124 | 10 | ≤ 0.25 | ≤ 0.25 | 4 | 4 | 1 | 1 | ≤ 0.25 | ≤ 0.25 |
| 7137 | 25 | 0.5 | 0.5 | ≤ 0.25 | ≤ 0.25 | ≤ 0.25 | ≤ 0.25 | ≤ 0.25 | ≤ 0.25 |
| 7153 | 203 | ≤ 0.25 | ≤ 0.25 | ≤ 0.25 | ≤ 0.25 | ≤ 0.25 | ≤ 0.25 | ≤ 0.25 | ≤ 0.25 |
| 7213 | 1 | ≤ 0.25 | ≤ 0.25 | ≤ 0.25 | ≤ 0.25 | ≤ 0.25 | ≤ 0.25 | ≤ 0.25 | ≤ 0.25 |
| 7251 | 32 | ≤ 0.25 | 0.5 | 2 | 2 | 1 | 1 | 1 | 1 |
| 7431 | 2 | 4 | 2 | 64 | 64 | 1 | 1 | 8 | 8 |
| 7446 | 963 | ≤ 0.25 | ≤ 0.25 | ≤ 0.25 | ≤ 0.25 | ≤ 0.25 | ≤ 0.25 | ≤ 0.25 | ≤ 0.25 |
| 7460 | 126 | ≤ 0.25 | ≤ 0.25 | 8 | 8 | ≤ 0.25 | ≤ 0.25 | ≤ 0.25 | ≤ 0.25 |
| 7521 | 415 | 4 | 2 | 64 | 64 | 2 | 2 | 32 | 32 |
| 7576 | 150 | ≤ 0.25 | ≤ 0.25 | ≤ 0.25 | ≤ 0.25 | ≤ 0.25 | ≤ 0.25 | ≤ 0.25 | ≤ 0.25 |
| 7690 | 23 | ≤ 0.25 | ≤ 0.25 | ≤ 0.25 | ≤ 0.25 | ≤ 0.25 | ≤ 0.25 | 0.5 | 0.5 |
| 7725 | 412 | ≤ 0.25 | ≤ 0.25 | ≤ 0.25 | ≤ 0.25 | ≤ 0.25 | ≤ 0.25 | ≤ 0.25 | ≤ 0.25 |
| 7735 | 78 | ≤ 0.25 | ≤ 0.25 | ≤ 0.25 | ≤ 0.25 | ≤ 0.25 | ≤ 0.25 | ≤ 0.25 | ≤ 0.25 |
| 10372 | 56 | ≤ 0.25 | ≤ 0.25 | ≤ 0.25 | ≤ 0.25 | ≤ 0.25 | ≤ 0.25 | ≤ 0.25 | ≤ 0.25 |
| 11224 | 1108 | 2 | 2 | 32 | 32 | 1 | 1 | 16 | 16 |
| 11663 | 136 | 0.5 | 0.5 | 1 | 1 | 2 | 2 | 16 | 16 |
| 11669 | 16 | 0.5 | 0.5 | ≤ 0.25 | ≤ 0.25 | ≤ 0.25 | ≤ 0.25 | 16 | 16 |
| 11695 | 229 | 2 | 2 | 0.5 | 0.5 | 1 | 1 | 32 | 32 |
| 11703 | 3 | 4 | 4 | 2 | 2 | 1 | 1 | 8 | 8 |
| 11816 | 374 | ≤ 0.25 | ≤ 0.25 | ≤ 0.25 | ≤ 0.25 | ≤ 0.25 | ≤ 0.25 | ≤ 0.25 | ≤ 0.25 |
| 14193 | 1 | 1 | 1 | 1 | 1 | 0.5 | 0.5 | 4 | 4 |
| 14237 | 1118 | ≤ 0.25 | ≤ 0.25 | 16 | 32 | ≤ 0.25 | ≤ 0.25 | 4 | 4 |
| 14427 | 622 | 0.5 | 0.5 | ≤ 0.25 | ≤ 0.25 | 0.5 | 0.5 | 16 | 16 |
| 15049 | 1 | 4 | 4 | 1 | 1 | 2 | 1 | 8 | 8 |
| 15070 | 403 | ≤ 0.25 | ≤ 0.25 | ≤ 0.25 | ≤ 0.25 | ≤ 0.25 | ≤ 0.25 | ≤ 0.25 | ≤ 0.25 |
| 15075 | 464 | ≤ 0.25 | ≤ 0.25 | ≤ 0.25 | ≤ 0.25 | ≤ 0.25 | ≤ 0.25 | ≤ 0.25 | ≤ 0.25 |
| 15088 | 107 | ≤ 0.25 | ≤ 0.25 | ≤ 0.25 | ≤ 0.25 | ≤ 0.25 | ≤ 0.25 | ≤ 0.25 | ≤ 0.25 |
| 15093 | 106 | ≤ 0.25 | ≤ 0.25 | ≤ 0.25 | ≤ 0.25 | ≤ 0.25 | ≤ 0.25 | ≤ 0.25 | ≤ 0.25 |
| 15129 | 1126 | 0.5 | 0.5 | 16 | 16 | ≤ 0.25 | ≤ 0.25 | 4 | 4 |
| 15574 | 85 | ≤ 0.25 | ≤ 0.25 | 1 | 1 | 0.5 | 0.5 | 4 | 8 |
| 16880 | 10 | ≤ 0.25 | ≤ 0.25 | 0.5 | 0.5 | 0.5 | 0.5 | 4 | 4 |
| 17493 | 632 | 2 | 2 | 32 | 32 | 1 | 1 | 8 | 16 |
| 19482 | 79 | 1 | 2 | 0.5 | 0.5 | 2 | 2 | 16 | 16 |
| 21660 | 250 | 1 | 1 | 64 | 64 | 0.5 | 0.5 | 8 | 8 |
| 21681 | 103 | 0.5 | 0.5 | 32 | 32 | 0.5 | 0.5 | 8 | 8 |
| 22112 | 193 | ≤ 0.25 | ≤ 0.25 | ≤ 0.25 | ≤ 0.25 | ≤ 0.25 | ≤ 0.25 | ≤ 0.25 | ≤ 0.25 |
| 23390 | 15 | 0.5 | 0.5 | ≤ 0.25 | ≤ 0.25 | ≤ 0.25 | ≤ 0.25 | 2 | 4 |
| 24008 | 2 | 2 | 4 | 64 | 64 | 1 | 2 | 8 | 8 |
| 24603 | 428 | ≤ 0.25 | ≤ 0.25 | ≤ 0.25 | ≤ 0.25 | ≤ 0.25 | ≤ 0.25 | ≤ 0.25 | ≤ 0.25 |
| 25547 | 2 | 8 | 8 | 1 | 1 | 4 | 4 | 32 | 32 |
| 29908 | 2 | 2 | 2 | 64 | 64 | 1 | 1 | 128 | 128 |
| 29999 | 240 | ≤ 0.25 | ≤ 0.25 | ≤ 0.25 | ≤ 0.25 | ≤ 0.25 | ≤ 0.25 | ≤ 0.25 | ≤ 0.25 |
| 30000 | 136 | 0.5 | 0.5 | 1 | 1 | 2 | 2 | 4 | 4 |
| 30885 | 40 | ≤ 0.25 | ≤ 0.25 | ≤ 0.25 | ≤ 0.25 | ≤ 0.25 | ≤ 0.25 | ≤ 0.25 | ≤ 0.25 |
| 30896 | 515 | ≤ 0.25 | ≤ 0.25 | 1 | 0.5 | ≤ 0.25 | ≤ 0.25 | ≤ 0.25 | ≤ 0.25 |
| 30909 | 32 | ≤ 0.25 | ≤ 0.25 | ≤ 0.25 | ≤ 0.25 | ≤ 0.25 | ≤ 0.25 | ≤ 0.25 | ≤ 0.25 |
| 30912 | 665 | ≤ 0.25 | ≤ 0.25 | ≤ 0.25 | ≤ 0.25 | ≤ 0.25 | ≤ 0.25 | ≤ 0.25 | ≤ 0.25 |
| 30945 | 48 | ≤ 0.25 | ≤ 0.25 | ≤ 0.25 | ≤ 0.25 | ≤ 0.25 | ≤ 0.25 | ≤ 0.25 | ≤ 0.25 |
| 31159 | 972 | ≤ 0.25 | ≤ 0.25 | ≤ 0.25 | ≤ 0.25 | ≤ 0.25 | ≤ 0.25 | ≤ 0.25 | ≤ 0.25 |
| 31196 | 20 | 1 | 1 | 0.5 | 1 | 0.5 | 0.5 | 16 | 16 |
| 31461 | 49 | 0.5 | 0.5 | ≤ 0.25 | ≤ 0.25 | ≤ 0.25 | 0.5 | 0.5 | 0.5 |
| 31468 | 154 | ≤ 0.25 | 0.5 | ≤ 0.25 | ≤ 0.25 | ≤ 0.25 | ≤ 0.25 | ≤ 0.25 | ≤ 0.25 |
| 31523 | 25 | ≤ 0.25 | ≤ 0.25 | ≤ 0.25 | ≤ 0.25 | ≤ 0.25 | ≤ 0.25 | ≤ 0.25 | ≤ 0.25 |
| 31915 | 1130 | ≤ 0.25 | ≤ 0.25 | ≤ 0.25 | ≤ 0.25 | ≤ 0.25 | ≤ 0.25 | ≤ 0.25 | ≤ 0.25 |
| 31937 | 498 | ≤ 0.25 | ≤ 0.25 | ≤ 0.25 | ≤ 0.25 | ≤ 0.25 | ≤ 0.25 | ≤ 0.25 | ≤ 0.25 |
| 31942 | 32 | ≤ 0.25 | ≤ 0.25 | ≤ 0.25 | ≤ 0.25 | ≤ 0.25 | ≤ 0.25 | ≤ 0.25 | ≤ 0.25 |
| 31947 | 32 | ≤ 0.25 | ≤ 0.25 | ≤ 0.25 | ≤ 0.25 | ≤ 0.25 | ≤ 0.25 | ≤ 0.25 | ≤ 0.25 |
| 32076 | 40 | ≤ 0.25 | ≤ 0.25 | ≤ 0.25 | ≤ 0.25 | ≤ 0.25 | ≤ 0.25 | ≤ 0.25 | ≤ 0.25 |
| 32104 | 25 | 2 | 2 | 8 | 8 | 8 | 8 | 4 | 4 |
| 32108 | 1 | 2 | 2 | 16 | 8 | 8 | 8 | 4 | 4 |
| 32142 | 155 | ≤ 0.25 | ≤ 0.25 | ≤ 0.25 | ≤ 0.25 | ≤ 0.25 | ≤ 0.25 | ≤ 0.25 | ≤ 0.25 |
| 32304 | 338 | ≤ 0.25 | ≤ 0.25 | ≤ 0.25 | ≤ 0.25 | ≤ 0.25 | ≤ 0.25 | ≤ 0.25 | ≤ 0.25 |
| 32797 | 1095 | ≤ 0.25 | ≤ 0.25 | ≤ 0.25 | ≤ 0.25 | ≤ 0.25 | ≤ 0.25 | ≤ 0.25 | ≤ 0.25 |
| 32842 | 1093 | ≤ 0.25 | ≤ 0.25 | ≤ 0.25 | ≤ 0.25 | ≤ 0.25 | ≤ 0.25 | ≤ 0.25 | ≤ 0.25 |
| 32865 | 94 | 0.5 | 0.5 | ≤ 0.25 | ≤ 0.25 | 0.5 | 0.5 | 8 | 16 |
| 32866 | 2 | 2 | 2 | 64 | 64 | 1 | 1 | 16 | 16 |
| 32875 | 388 | ≤ 0.25 | ≤ 0.25 | ≤ 0.25 | ≤ 0.25 | ≤ 0.25 | ≤ 0.25 | ≤ 0.25 | ≤ 0.25 |
| 32892 | 307 | 0.5 | 0.5 | ≤ 0.25 | ≤ 0.25 | 0.5 | 0.5 | ≤ 0.25 | ≤ 0.25 |
| 32915 | 2 | 2 | 2 | 32 | 32 | 1 | 1 | 8 | 8 |
| 337038 | 636 | 2 | 2 | 0.5 | 0.5 | 1 | 1 | 8 | 4 |
| 351162 | 412 | ≤ 0.25 | ≤ 0.25 | ≤ 0.25 | ≤ 0.25 | ≤ 0.25 | ≤ 0.25 | ≤ 0.25 | ≤ 0.25 |
| 351524 | 113 | 0.5 | 0.5 | ≤ 0.25 | ≤ 0.25 | 0.5 | 0.5 | ≤ 0.25 | ≤ 0.25 |
| 423159 | 2 | 2 | 2 | 1 | 1 | 2 | 2 | 32 | 32 |
| 480561 | 25 | ≤ 0.25 | ≤ 0.25 | ≤ 0.25 | ≤ 0.25 | ≤ 0.25 | ≤ 0.25 | 4 | 4 |
| 480622 | 215 | ≤ 0.25 | ≤ 0.25 | 2 | 2 | ≤ 0.25 | ≤ 0.25 | 4 | 2 |
| 489669 | 2 | 0.5 | 0.5 | ≤ 0.25 | ≤ 0.25 | ≤ 0.25 | ≤ 0.25 | 8 | 4 |
| 489678 | 19 | ≤ 0.25 | ≤ 0.25 | ≤ 0.25 | ≤ 0.25 | ≤ 0.25 | ≤ 0.25 | 32 | 16 |

Abbreviation: MRSN: Multidrug-Resistant Organism Repository and Surveillance Network and MIC: minimum inhibitory concentration. A: Replication 1,B: Replication 2


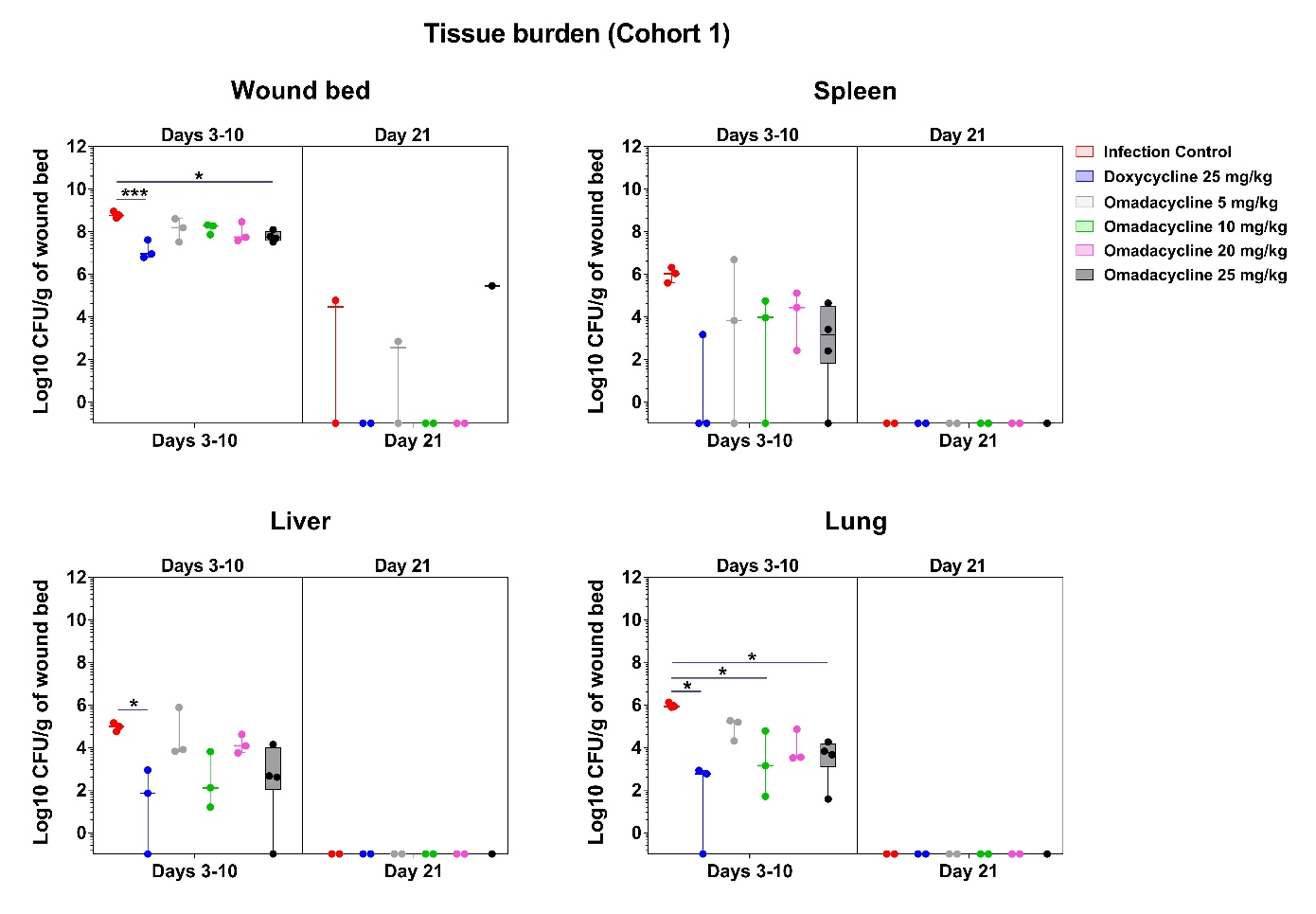


**Figure S1. Cohort 1: Bacterial burden in tissues of *A. baumannii*-infected mice treated with either doxycycline or omadacycline.**

Infection control, doxycycline 25 mg/kg, or omadacycline 5, 10, 20 and 25 mg/kg were administered *via* IP injection on days 0-5. The Box-and-Whisker plot shows the medians and interquartile ranges with 95% Confidence interval. Each dot represents data from one mouse. P-values are shown for significant differences relative to infection control within the same assay days. One-way ANOVA followed by Dunnett’s multiple comparisons test; *, p < 0.05; **, p < 0.01; ***, p < 0.001.


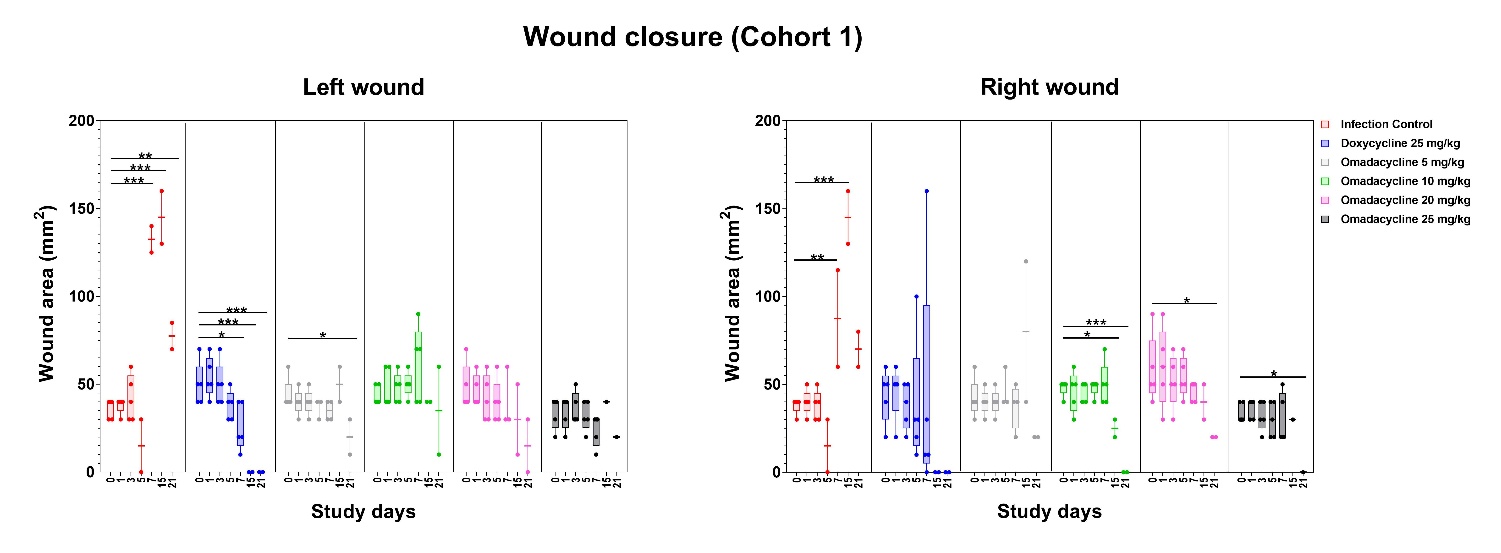


**Figure S2. Cohort 1: Time courses of wound area size of *A. baumannii*-infected mice treated with either doxycycline or omadacycline.**

Infection control, doxycycline 25 mg/kg, or omadacycline 5, 10, 20 and 25 mg/kg were administered *via* IP injection on days 0-5. The Box-and-Whisker plot shows the medians and interquartile ranges with 95% CI. Each dot represents data from each mouse. P-values are shown significant differences relative to day 0 within the same group. One-way ANOVA followed by Dunnett’s multiple comparisons test; *, p < 0.05; **, p < 0.01; ***, p < 0.001.
